# Supplementary material for: Seamless and non-destructive monitoring of extracellular microRNAs during cardiac differentiation from human pluripotent stem cells
Source: Stem Cell Reports. 2023 Sep 21;18(10):1925–39. doi: 10.1016/j.stemcr.2023.08.011 (PMC10656301; doi:10.1016/j.stemcr.2023.08.011)
Supplement: Document S1. Figures S1–S6 and supplemental experimental procedures [file mmc1.pdf]

Stem Cell Reports, Volume *18*

Supplemental Information

Seamless and non-destructive monitoring of extracellular microRNAs during cardiac differentiation from human pluripotent stem cells

Otoya Sekine, Sayaka Kanaami, Kanako Masumoto, Yuki Aihara, Yuika Morita-Umei, Hidenori Tani, Yusuke Soma, Tomohiko C. Umei, Kotaro Haga, Taijun Moriwaki, Yujiro Kawai, Masatoshi Ohno, Yoshikazu Kishino, Hideaki Kanazawa, Keiichi Fukuda, Masaki Ieda, and Shugo Tohyama

Figure S1

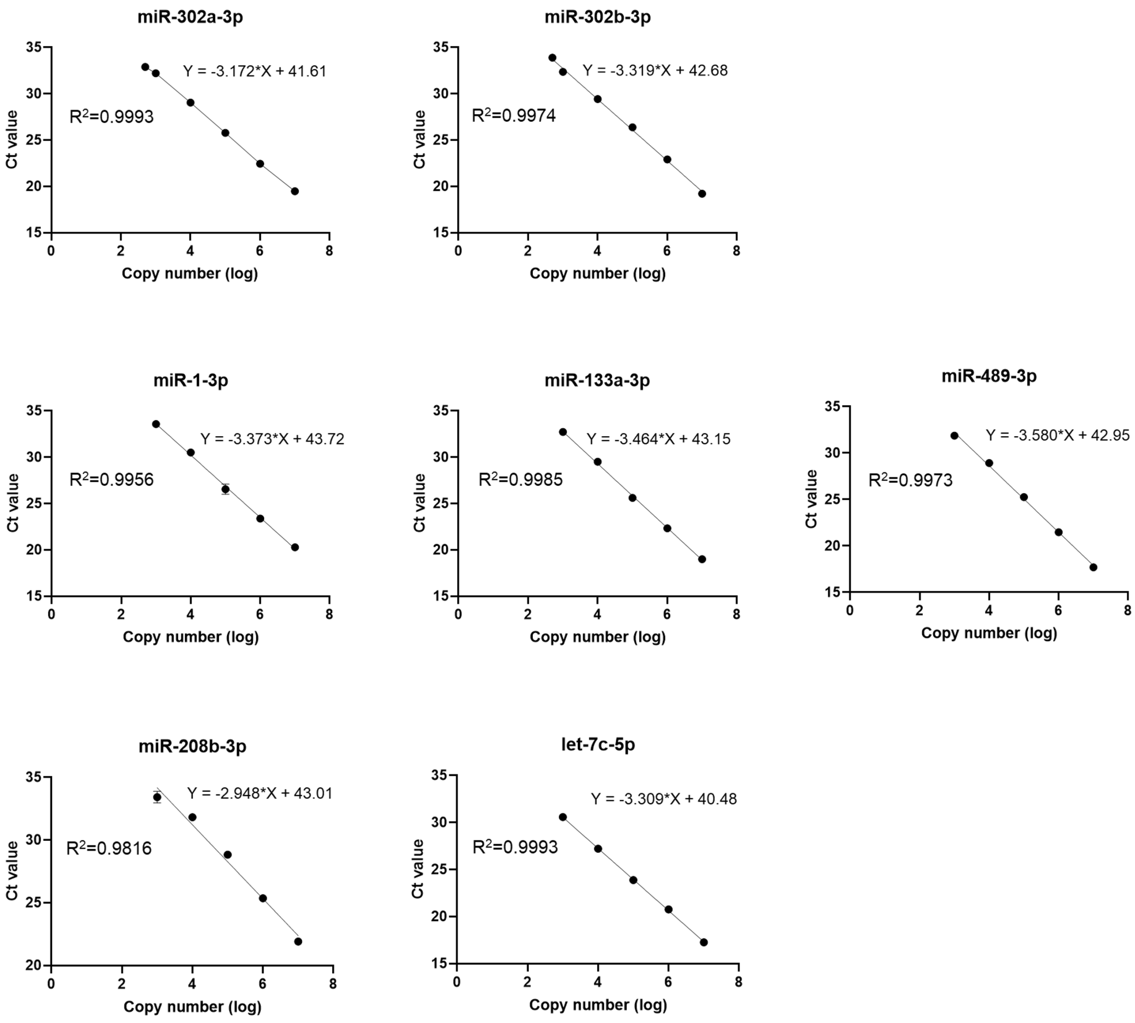

Figure S1. Correlation between the number of copies of each synthetic miRNA and the Ct value in RT-qPCR, related to Figure 1.

The linear dynamic changes between copy numbers and Ct values were 1 × 10^3–1 × 10^7 copies for synthetic miR-489-3p, miR-1-3p, miR-133a-3p, miR-208b-3p, and let-7c-5p and from 500–1 × 10^7 copies for synthetic miR-302a-3p and synthetic miR-302b-3p. R^2: Coefficient of determination.

Figure S2

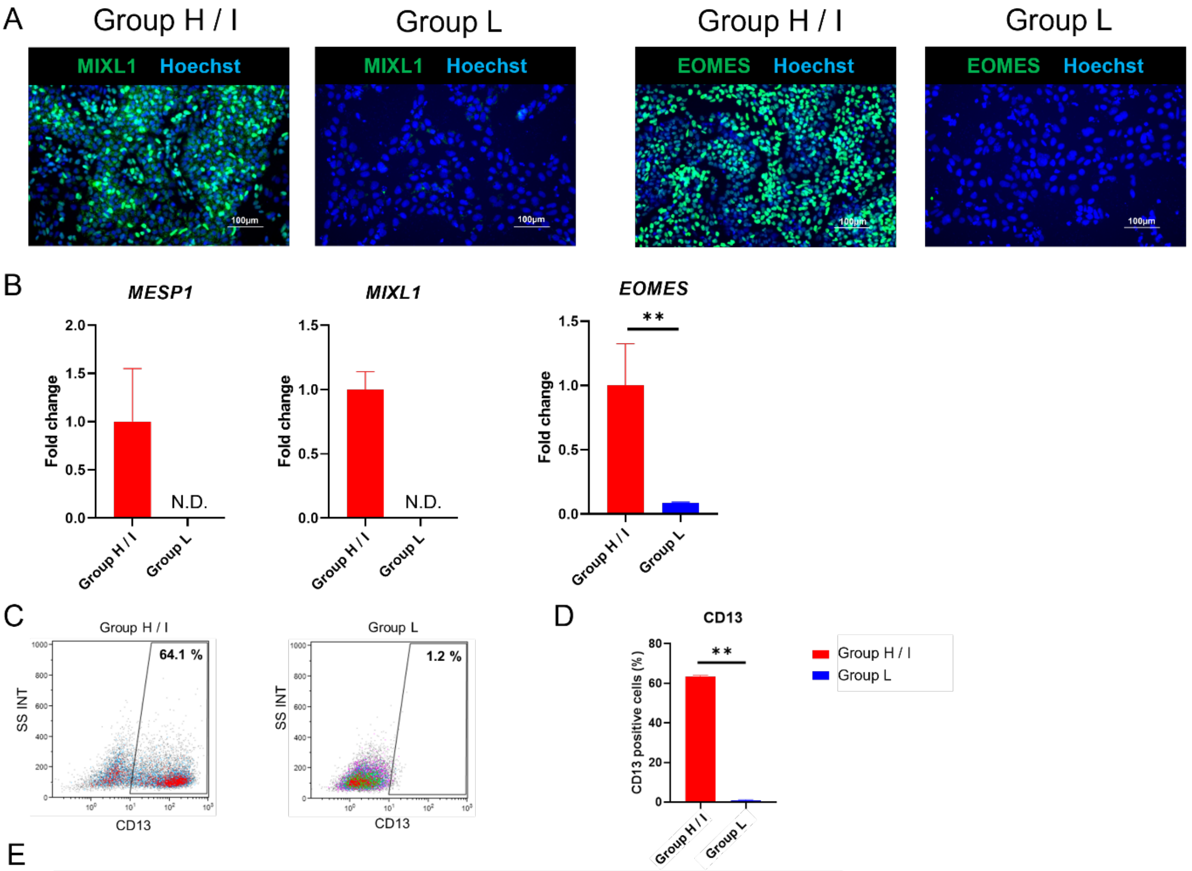

E

Sample Name	Self-renewal	Ectoderm	Mesoderm	Endoderm
Group H / I	-2.86	0.36	1.95	0.52
Group L	-1.44	0.09	-0.61	-1.49

upregulated						Downregulated
X > 1.5	1.0 < X ≤ 1.5	0.5 < X ≤ 1.0	-0.5 ≤ X ≤ 0.5	-1.0 ≤ X < -0.5	-1.5 ≤ X < -1.0	X < -1.5

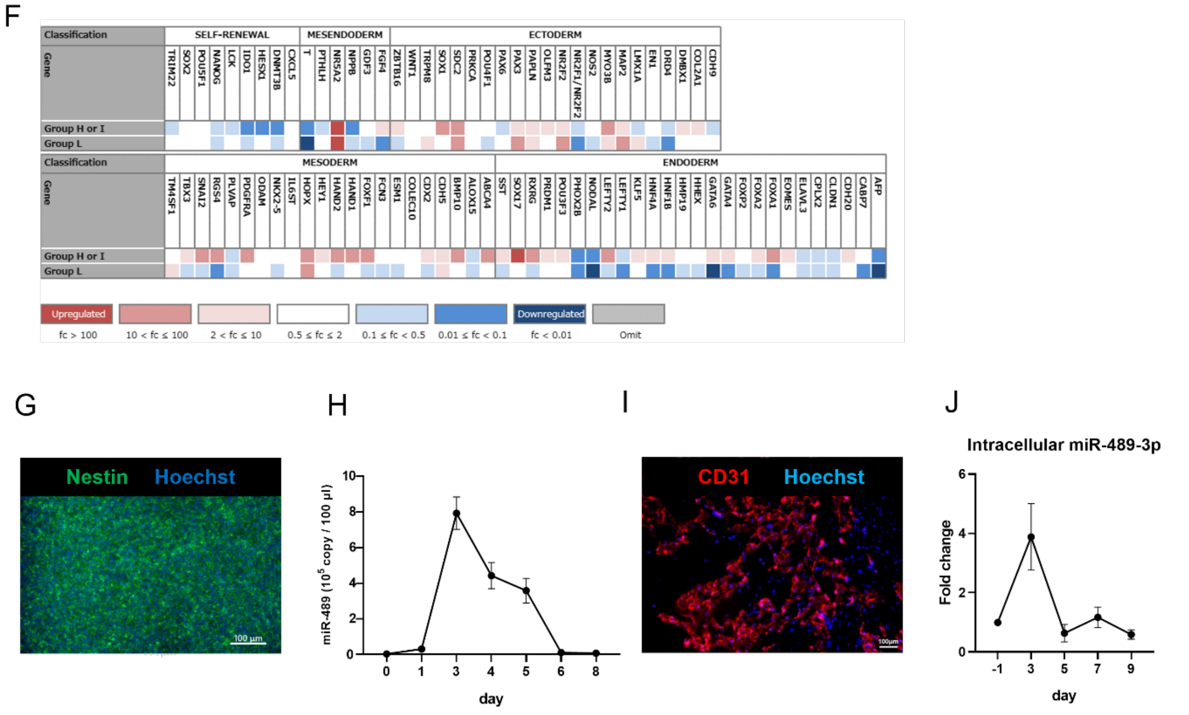

Figure S2. Verification of the usefulness of miR-489-3p for monitoring mesoderm differentiation, related to Figure 2.

(A) Immunostaining for MIXL1 or EOMES (green) and Hoechst (blue) in Groups H / I and L on day 3 differentiated from hiPSCs (253G4). Scale bar, 100 μm.

(B) RT-qPCR analysis of the relative expression levels of *MESP1*, *MIXL1*, and *EOMES* in Groups H / I and L on day 3 (n = 4 independent experiments for 253G4 cell lines).

(C and D) Flow cytometry analysis of CD13-positive cells in Groups H / I and L on day 3 (n = 3 independent experiments for the 253G4 cell line).

(E) Scorecard analysis for Group H / I and L on day 3 differentiated from hiPSCs (253G4). Algorithmic scores were generated by Scorecard analysis based on the expression of 96 genes per sample. Scores are a statistical comparison of the expression profile of the sample to that of the undifferentiated reference set.

(F) Heatmaps showing gene expression associated with trilineage differentiation and pluripotency in each sample. Colors correlate with the fold change in expression of the indicated gene relative to the undifferentiated reference set.

(G) Immunostaining for Nestin (green) and Hoechst (blue) in Neural stem cells on day 3 differentiated from hiPSCs (253G4). Scale bar, 100 μm.

(H) miR-489-3p levels in the culture supernatant during endothelial cell differentiation from hiPSCs (n = 3 independent experiments for 253G4 cell lines).

(I) Immunostaining for CD31 (red) and Hoechst (blue) in endothelial cells differentiated from hiPSCs (253G4) on day 8. Scale bar, 100 μm.

(J) miR-489-3p expression in the cells during mesoderm/cardiac differentiation from hiPSCs (n = 4 independent experiments for 253G4 cell lines).

Data are presented as mean ± SD; $*p < 0.05$; $**p < 0.01$. All p values are determined with a ratio-paired *t*-test.

Figure S3

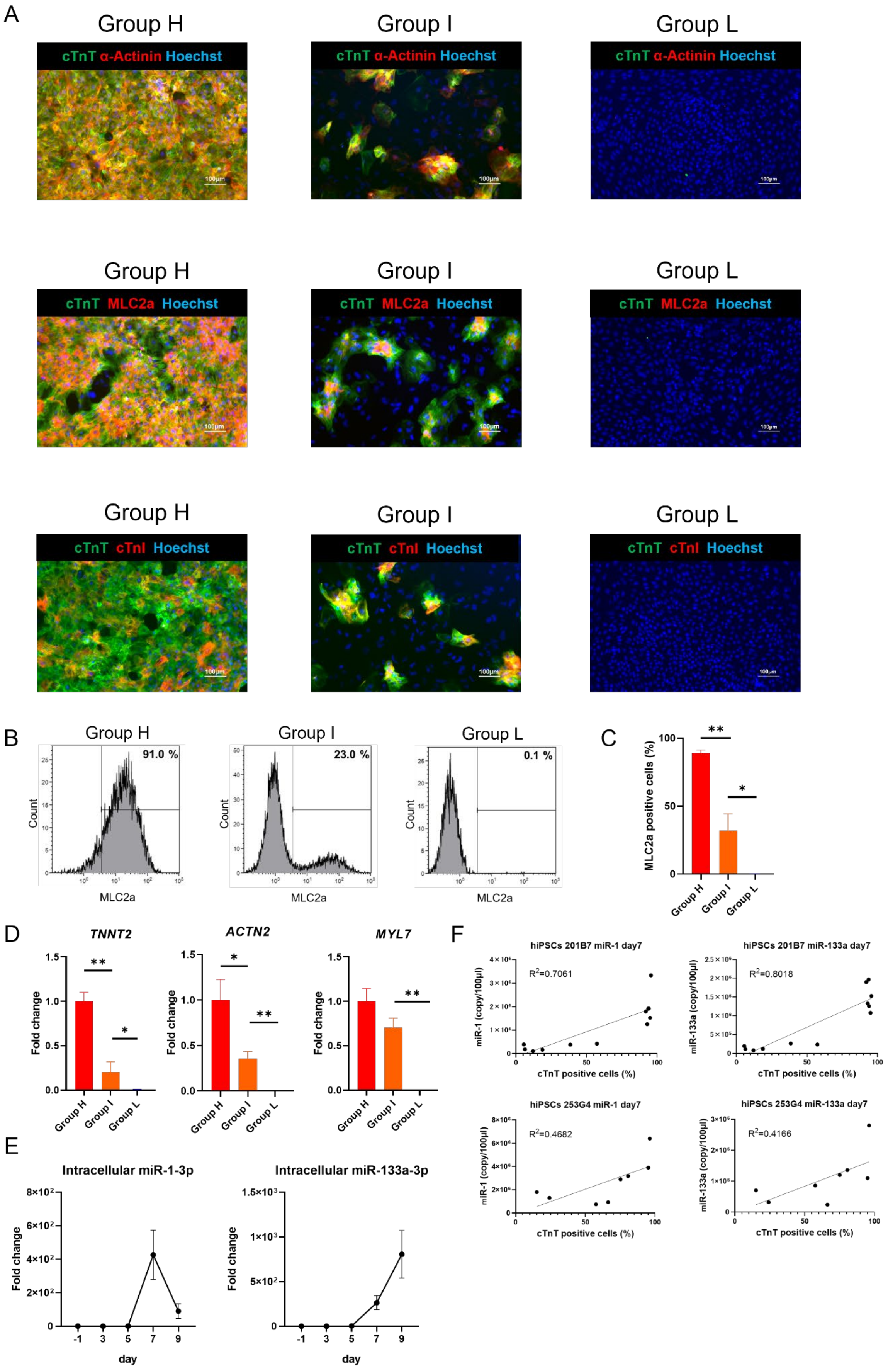
A
Group H
Group I
Group L
cTnT α-Actinin Hoechst
cTnT MLC2a Hoechst
cTnT cTnI Hoechst
100µm
B
91.0 %
23.0 %
0.1 %
Count
MLC2a
C
MLC2a positive cells (%)
100
50
0
**
*
D
TNNT2
ACTN2
MYL7
Fold change
1.5
1.0
0.5
0.0
E
Intracellular miR-1-3p
Intracellular miR-133a-3p
Fold change
day
-1
3
5
7
9
F
hiPSCs 201B7 miR-1 day7
hiPSCs 201B7 miR-133a day7
hiPSCs 253G4 miR-1 day7
hiPSCs 253G4 miR-133a day7
R²=0.7061
R²=0.8018
R²=0.4682
R²=0.4166
miR-1 (copy/100µl)
miR-133a (copy/100µl)
cTnT positive cells (%)

Figure S3. Verification of the usefulness of miR-1-3p and miR-133a-3p for monitoring cardiac differentiation, related to Figure 3.

(A) Immunostaining for α-Actinin, MLC2a or cTnI (red), cTnT (green), and Hoechst (blue) in Groups H, I, and L on day 10 differentiated from hiPSCs (253G4). Scale bar, 100 μm.

(B and C) Flow cytometry analysis for MLC2a-positive cells in Groups H, I, and L on day 10 (n = 4 independent experiments for the 253G4 cell line).

(D) RT-qPCR analysis of the relative gene expression of *TNNT2*, *ACTN2,* and *MYL7* in Groups H, I, and L on day 10 (n = 4 independent experiments for 253G4 cell lines).

(E) miR-1-3p and miR-133a-3p expression in the cells during cardiac differentiation from hiPSCs (n = 4 independent experiments for 253G4 cell lines).

(F) Correlation between the percentage of cardiac troponin T-positive cells on day 10 and the amount of miR-1,133a-3p in the culture supernatant on day 7. R^2: Coefficient of determination. Data are presented as mean ± SD; *$p < 0.05$; **$p < 0.01$. All p values are determined with a ratio-paired *t*-test.

Figure S4

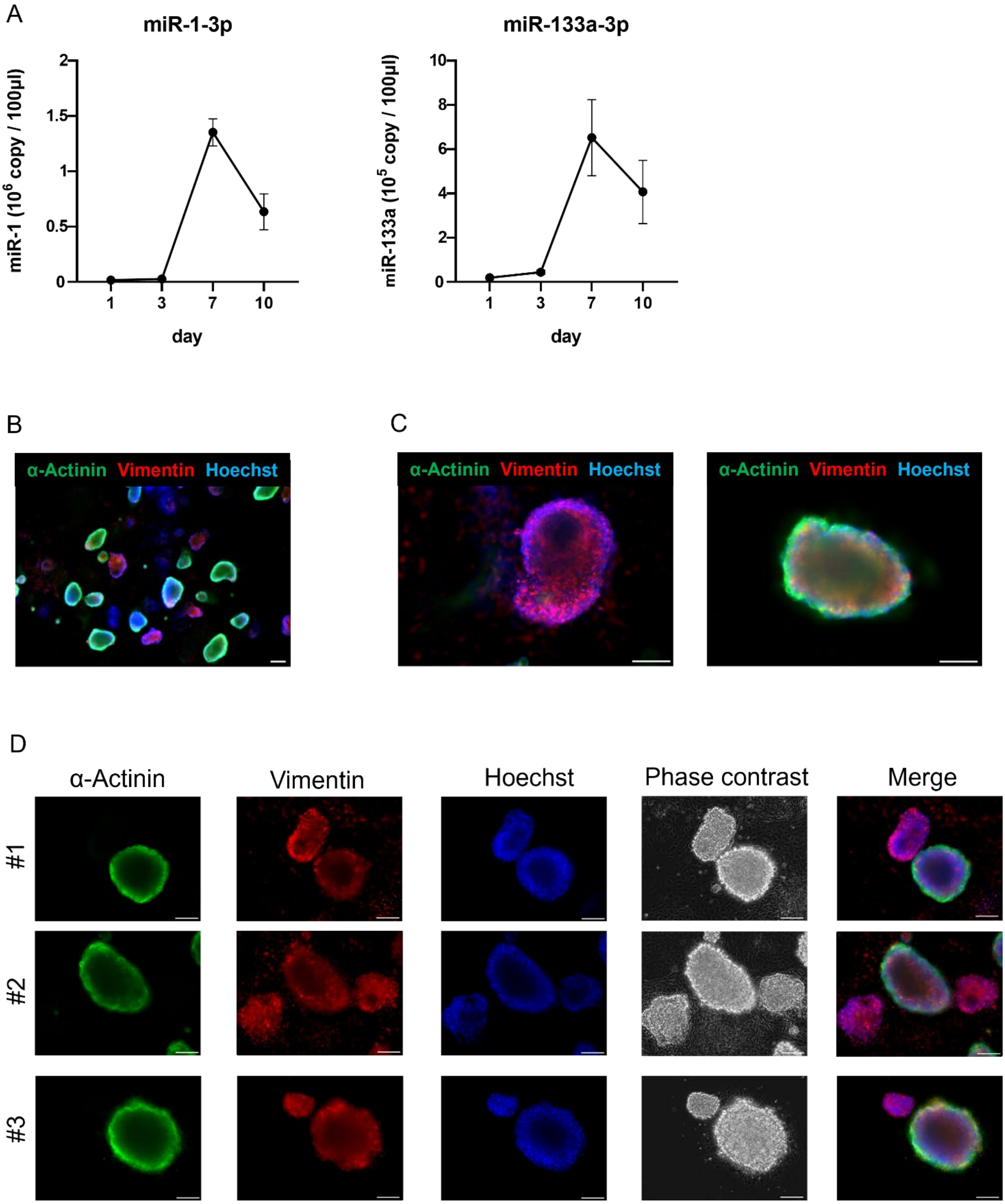

A
miR-1-3p
miR-1 (10^6 copy / 100µl)
2
1.5
1
0.5
0
1
3
7
10
day
miR-133a-3p
miR-133a (10^5 copy / 100µl)
10
8
6
4
2
0
1
3
7
10
day
B
α-Actinin Vimentin Hoechst
C
α-Actinin Vimentin Hoechst
α-Actinin Vimentin Hoechst
D
α-Actinin
Vimentin
Hoechst
Phase contrast
Merge
#1
#2
#3

Figure S4. Application of cardiac differentiation monitoring by measuring miR-1-3p and miR-133a-3p levels in culture supernatant to 3D culture, related to Figure 3.

(A) miR-1-3p and miR-133a-3p levels in the culture supernatant during cardiac differentiation from hiPSC-CMs in 3D culture method using bioreactors (n = 3 independent experiments for 253G4 cell lines).

(B) Immunostaining of multiple cell aliquots differentiated from hiPSCs (253G4) with α-Actinin (Green), Vimentin (red), and Hoechst (blue) collected when cultured in the same bioreactor for 3D culture. Scale bar, 200 μm.

(C and D) Enlarged images of the part of Figure S2. Scale bar, 100 μm.

Data are presented as mean ± SD.

Figure S5

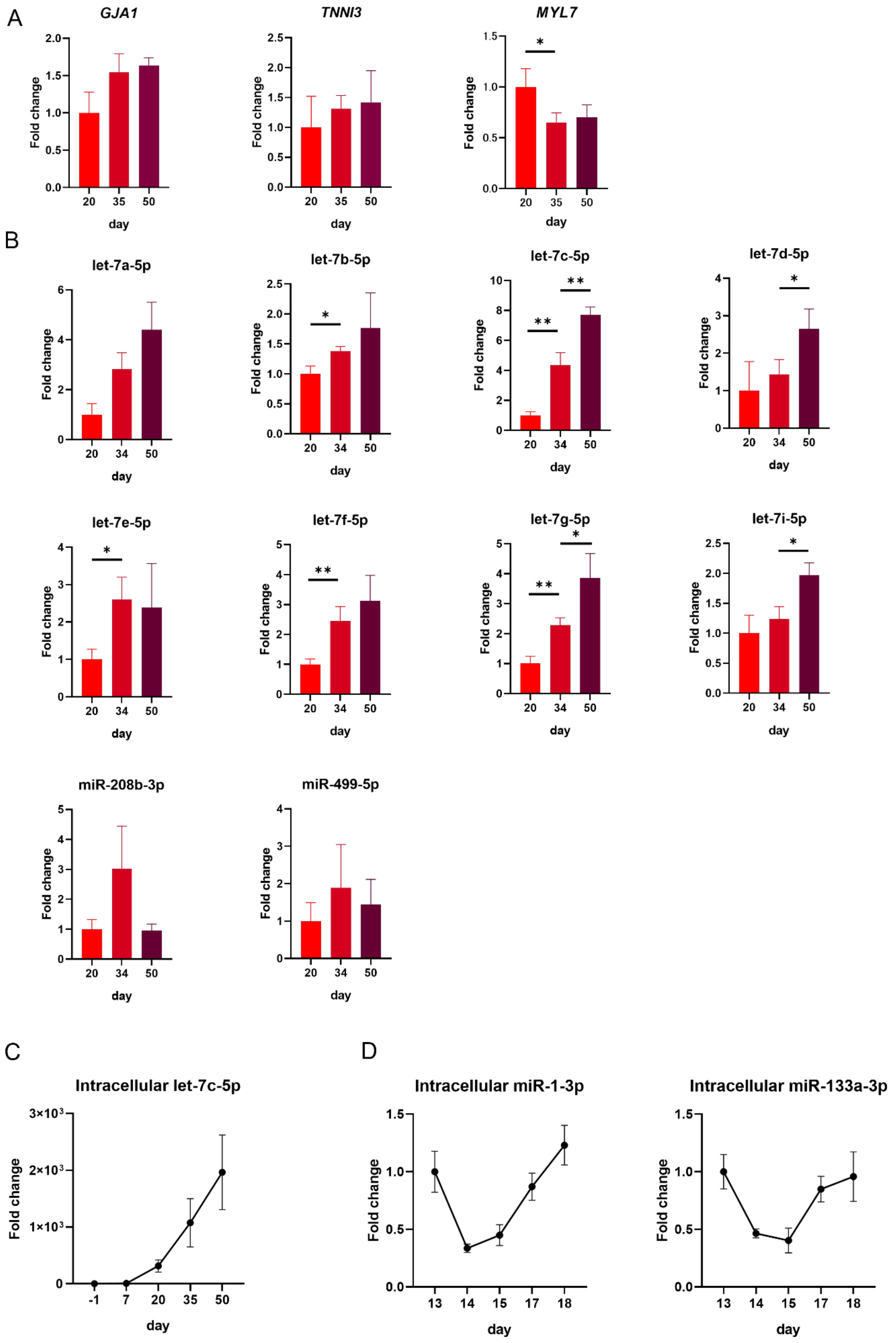
A
GJA1
TNNI3
MYL7
Fold change
day
20
35
50
*
B
let-7a-5p
let-7b-5p
let-7c-5p
let-7d-5p
let-7e-5p
let-7f-5p
let-7g-5p
let-7i-5p
miR-208b-3p
miR-499-5p
Fold change
day
20
34
50
*
**
C
Intracellular let-7c-5p
Fold change
3×10³
2×10³
1×10³
0
-1
7
20
35
50
day
D
Intracellular miR-1-3p
Intracellular miR-133a-3p
Fold change
1.5
1.0
0.5
0.0
13
14
15
17
18
day

Figure S5. miRNAs that significantly increase in the supernatant during cardiac maturation, related to Figure 5.

(A) RT-qPCR analysis of the relative expression of *MYL7*, *GJA1*, and *TNNI3* in hiPSC-CMs on days 20, 35, and 50 measured by (n = 4 independent experiments for 253G4 cell lines).

(B) The fold change in the level of each miRNA in the culture supernatant during long-term culture of hiPSC-CMs on days 20, 34, and 50 (n = 3 independent experiments for the 253G4 cell lines).

(C) let-7c-5p expression in the cells during cardiac differentiation and maturation from hiPSCs (n = 4 independent experiments for 253G4 cell lines).

(D) miR-1-3p and miR-133a-3p expression in the cells during cardiac purification step (n = 3 independent experiments for 253G4 cell lines).

Data are presented as mean ± SD; $*p < 0.05$; $**p < 0.01$. All p-values are determined with a ratio-paired *t*-test.

Figure S6

A

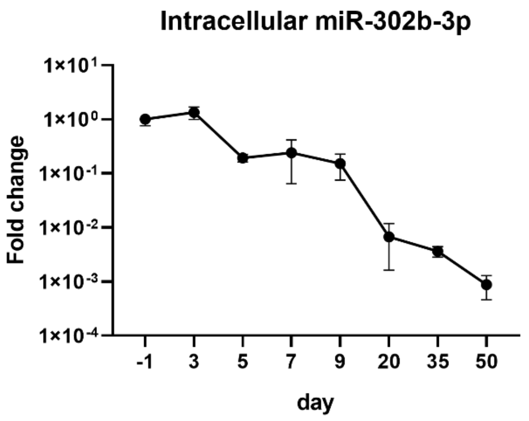

B

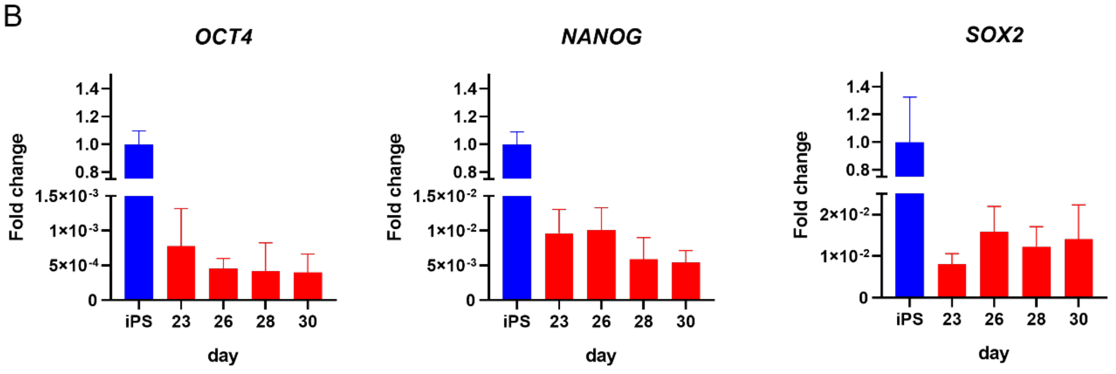

C

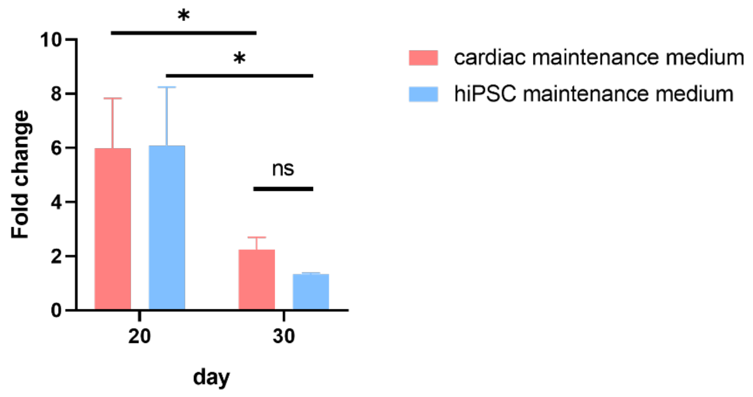

Figure S6. **Verification of the usefulness of miR-302b-3p for the detection of residual undifferentiated hiPSCs in hiPSC-CM cultures, related to Figure 6.**

(A) miR-302b-3p expression in the cells during cardiac differentiation and maturation from hiPSCs (n = 4 independent experiments for 253G4 cell lines).

(B) RT-qPCR analysis of the relative expression of *OCT4, NANOG,* and *SOX2* in pure hiPSCs and hiPSC-CMs on day 23, 26, 28, and 30 (n = 3 independent experiments for 253G4 cell lines).

(C) Intracellular expression of miR-302b-3p in hiPSC-CMs cultured under cardiac maintenance conditions and hiPSC maintenance conditions.

Data are presented as mean ± SD; *p < 0.05; **p < 0.01. All p-values are determined with a ratio-paired *t*-test.

Experimental procedures

Cell lines

Human induced pluripotent stem cell (hiPSC) lines (253G4 and 201B7) were obtained from the Center for iPS Cell Research and Application (CiRA), Kyoto University. Clinical-grade HLA homozygous hiPSC line (QHJI14s04) was also obtained from CiRA (Yoshida et al., 2023). The HEK293T cell line was obtained from RIKEN BioResource Research Center.

Maintenance of hiPSC lines

hiPSCs were maintained on Matrigel (Corning, 354230) or iMatrix511 (Nippi, NP892-011) coated plates in animal-free and chemically-defined hPSC maintenance medium (StemFit AS103C, Ajinomoto) (Kameda et al., 2022; Someya et al., 2021; Tohyama et al., 2017). Cells were routinely passaged every 5 or 6 days. After being washed with D-PBS (FUJIFILM Wako Pure Chemical, 045-29795), cells were dissociated using StemPro Accutase (Thermo Fisher Scientific, A1110501). Dissociated cells were collected in clinical-grade hiPSC maintenance medium, StemFit AS103C, with 10 μM of CultureSure Y-27632 (FUJIFILM Wako Pure Chemical, 034-24024). Following centrifugation (300 × g for 4 min), supernatant aspiration, and addition of hPSC maintenance medium with Y-27632, the number of cells was counted with a Vi-CELL XR (Beckman Coulter), and about 1×10^5 cells were then seeded onto a Matrigel- or iMatrix511-coated 10 cm plate. Media were changed every other day using the hPSC maintenance medium.

Maintenance of HEK293T cells

HEK293T cells were maintained in DMEM (Gibco, 11885) supplemented with 10% fetal bovine serum (FBS) (Biowest, S1560-500) on 0.1% gelatin-coated plates. HEK293T cells were routinely passaged every 3 or 4 days using a similar procedure to the passaging of hiPSCs mentioned above but using DMEM plus 10% FBS.

Production of hiPSC-CMs and supernatant sampling

hiPSC-derived cardiomyocytes (hiPSC-CMs) were generated as previously described (Tanosaki et al., 2022; Tohyama *et al.*, 2017; Tohyama et al., 2016). Briefly, 4 days before day 0 hiPSCs were passaged onto Matrigel- or iMatrix511-coated plates and incubated with clinical-grade hPSC maintenance medium for 4 days to reach 90–100% confluent. On day 0, cells were washed with D-PBS and incubated in animal-free and chemically-defined cardiac differentiation medium (StemFit AS301, Ajinomoto) or RPMI1640 (FUJIFILM

Wako Pure Chemical, 189-02025) supplemented with 2% B27 supplement without insulin (Thermo Fisher Scientific, A1895601), 6 μM CHIR99021 (FUJIFILM Wako Pure Chemical, 034-23103), and 1 ng/mL bone morphogenic protein 4 (BMP4) (R&D Systems, 314-BP) for 1 day. On day 1, cells were washed with D-PBS and incubated in clinical-grade StemFit AS301 or RPMI1640 supplemented with B27 supplement without insulin. The same procedure was followed on day 3 but 5 μM IWR-1 (Sigma-Aldrich, I0161-25MG) was added. On day 5 or 6, the cells were washed and the medium was replaced with clinical-grade StemFit AS301 or RPMI1640 supplemented with B27 supplement without insulin. On day 7, cells were incubated in MEMα (Thermo Fisher Scientific, 12571-048) supplemented with 5% FBS (SAFC Bioscience Inc.,12003C or Biowest, S1560-500) and 2 mM sodium pyruvate (Sigma, S8636-100ML) for maintenance of hiPSC-CMs. On days 8–10, the cells were washed with D-PBS and dissociated using 0.25% Trypsin/1 mM EDTA. Cells were collected and resuspended in MEMα plus 5% FBS and counted using Vi-CELL (Beckman Coulter). Cells were plated in plates coated with collagen type I (AGC TECHNO GLASS, 4020-010) or iMatrix-221 (Nippi, NP892-061). On day 13, the culture medium was replaced with glucose and glutamine-free medium supplemented with 4 mM lactate, animal-free and chemically-defined cardiac purification medium (StemFit AS501, Ajinomoto) for metabolic selection (Tohyama *et al.*, 2016; Tohyama et al., 2013). Four days after replacing the medium with clinical-grade cardiac purification medium (StemFit AS501, Ajinomoto), the culture medium was replaced by MEMα plus 5% FBS. On day 17 or later, cardiomyocytes were used for experiments. In the experiment to measure the amount of miR-489-3p, miR-1-3p, and miR-133a-3p secreted during the differentiation process, medium exchange and supernatant collection were performed from day −1 to day 9. In this differentiation process, we set three groups based on mesoderm and cardiac differentiation efficiency, high (Group H), intermediate (Group I), and low (Group L). In Group H, we produced hiPSC-derivatives with CHIR99021/BMP4 and IWR1. In Group I, we produced them with CHIR99021/BMP4 and without IWR1. In Group L, we produced them without CHIR99021/BMP4. In this experiment, the supernatant was collected at the time of the medium exchange. In collecting supernatant samples for miRNA microarray during the differentiation process, medium exchange and supernatant collection were performed as shown in **Figure 1A**. For the 3D culture method, 4×10^6 hiPSCs in 25 mL of hPSC maintenance medium with 10 μM Y-27632 were incubated in a single-use bioreactor (Biott, BWV-S03A) rotating at 60 rpm for 4 days before day 0, and hiPSC-CMs were subsequently induced following the same protocol as the 2D culture system.

Production of endothelial cells from hiPSCs

Briefly, 4 days before day 0 hiPSCs were passaged onto Matrigel- or iMatrix511-coated plates and incubated with hPSC maintenance medium for 4 days to reach 90–100% confluence. On day 0, cells were washed with D-PBS and incubated in RPMI1640 supplemented with 2% B27 supplement without insulin, 2 mM GlutaMAX (Gibco, 35050061), 6 µM CHIR99021, and 10 ng/mL BMP4 for 1 day. On day 1, cells were washed with D-PBS and incubated in RPMI1640 supplemented with B27 supplement without insulin, 2 mM GlutaMAX, and 10 µg/ml bFGF (Ajinomoto). The same procedure was followed on day 3. On day 4, the medium was replaced with RPMI1640 supplemented with B27 supplement without insulin, GlutaMAX, and 100 µg/mL VEGF (PeproTech, 100-20). On day 5, the cells were washed with D-PBS and dissociated using 0.25% Trypsin/1 mM EDTA and passaged onto Matrigel- or iMatrix511-coated plates and incubated with RPMI1640 supplemented with B27 supplement without insulin, GlutaMAX and 100 µg/mL VEGF. On days 6 and 8, the medium was replaced with the same as before.

Production of neural cells from hiPSCs

Ectodermal cells (Neural cells) were induced from hiPSCs using STEMdiff SMADi Neural Induction Kit (STEMCELL ST-08581). Briefly, hiPSCs were passaged onto Matrigel- or iMatrix511-coated plates at the density of 2.0-2.5 $\times 10^5$ cells/cm^2. The cells were then incubated with attached medium supplemented with 10 µM Y27632. The medium was changed to the same medium without Y27632 the next day and 2 days later. After 3 days, the cells were fixed in 4% paraformaldehyde (Muto Pure Chemicals, 33111) for 15–20 min and Immunostaining with Nestin was performed following the immunostaining protocol described below.

Immunofluorescence staining

Cells were washed once with D-PBS and fixed in 4% paraformaldehyde (Muto Pure Chemicals, 33111) for 15–20 min. Cells were then washed with D-PBS, and treated with 0.1% Triton X (Sigma-Aldrich, T9284) for 5–15 min. Cells were then washed with PBS supplemented with 0.05% Tween-20 (Thermo Fisher Scientific, 28360) (PBS-T) and treated with a blocking solution of ImmunoBlock (KAC, CTKN001) for 1 h at room temperature or overnight at 4°C. Following primary incubation with a primary antibody diluted in ImmunoBlock overnight at 4°C, cells were washed twice with D-PBS and co-incubated with a secondary antibody diluted in ImmunoBlock at room temperature for 2 h. Cells were washed twice more with D-PBS and treated with 5 µg/mL Hoechst 33342 (Thermo Fisher Scientific, H3570) at room temperature for 1 h. Finally, cells were washed twice with D-PBS and incubated in ImmunoBlock until staining was examined using BZ-X710

(Keyence). Immunofluorescence staining of cultured cells was performed using the following primary antibodies; anti-Brachyury T (Abcam, ab209665;1:200), anti-MIXL1 (Proteintech Group Inc, 22772-1-AP; 1:100), anti-EOMES (Abcam, ab216870; 1:100), anti-CD31(Abcam, ab28364; 1:50), and anti-Nestin (Abcam, ab 105389; 1:200), anti-OCT-3/4 (Abcam, ab200834; 1:200), anti-SSEA4 (Chemicron, MAB4304; 1:100), anti-cardiac Troponin T (Abcam, ab45932; 1:800, or Neomarkers, MS-295-P; 1:200), anti-cardiac Troponin I (Abcam, ab52862; 1:100), anti-α-Actinin (Abcam, ab137346; 1:100), anti-N-cadherin (Invitrogen, 333900; 1:100), anti-Connexin 43 (Sigma-Aldrich, C6219; 1:100), anti-MLC2a (Synaptic Systems C156F5 311011; 1:100), anti-MLC2v (Abcam, Cambridge, USA, ab 79935; 1:100), and anti-Vimentin (Abcam, ab8069; 1:1000) and using the following secondary antibodies: anti-rabbit IgG or anti-mouse IgG conjugated with Alexa Fluor 488, Alexa Fluor 546, or Alexa Fluor 594 (all from Thermo Fisher Scientific; 1:200).

Colony formation assay for detection of residual undifferentiated hPSCs

The colony formation assay (CFA) was performed to confirm the absence of undifferentiated hiPSCs in the hiPSC-CMs used for experiments, following previous reports (Tano et al., 2014; Tohyama *et al.*, 2016). A total of 3×10^6 HEK293T cells or hiPSC-CMs were seeded onto 6-well plates coated with iMatrix-511; the group without hiPSC contamination served as a negative control, the group contaminated with 0.001% hiPSCs served as a positive control, and the sample to be verified was also seeded 3×10^6 cells/well. All groups were cultured in hPSC maintenance medium (StemFit AS103C, Ajinomoto) for 2 days. Subsequently, they were fixed with 4% paraformaldehyde, and OCT4 and SSEA4-positive colonies were detected via immunostaining. If a colony was detected in the positive control and not detected in the sample as well as the negative control, the contamination rate of residual undifferentiated hiPSCs in the sample is less than 0.001%.

Flow cytometry analysis

Cells were washed with D-PBS and incubated with 0.25% Trypsin/1 mM EDTA or StemPro Accutase (Thermo Fisher Scientific) for 5 min. Following collection of the dissociated cells, they were centrifuged (300 g × 4 min), the supernatant was aspirated and the cells were fixed by adding 4% paraformaldehyde for 20 min. D-PBS was then added to the cells, and following centrifugation (300 × g for 4 min) and supernatant aspiration, 0.1% Triton X was added for 5–15 min. Then, the cells were washed with PBS-T as mentioned above, dispensed into aliquot tubes with 100 μL of a solution containing ImmunoBlock with 2 μL of the specific antibody added

to each tube, and left in the dark for 15–20 min. Cells were washed with PBS, diluted in ImmunoBlock, and analyzed using a Gallios Flow Cytometer (Beckman Coulter). Antibodies used were: anti-REA Control FITC, human (Miltenyi Biotec, 130-118-354), anti-REA Control APC, human (Miltenyi Biotec, 130-118-546), anti-cardiac Troponin T (cTnT), human (Miltenyi Biotec, 130-119-575), anti-cardiac MLC2a, human (Miltenyi Biotec, 130-118-546), IgG1-PE Isotypic Control (IMMUNOTECH SAS, A07796), and anti-CD13-PE (IMMUNOTECH SAS, A07762).

RNA extraction and quantitative RT-PCR analysis

Total RNA samples were extracted using ReliaPrep™ RNA Cell Miniprep System (Promega) according to the manufacturer's instructions. The concentration and purity of the RNA were measured using an ND-1000 spectrophotometer (Nanodrop) and the cDNA was synthesized using the Superscript First Strand Synthesis System (Invitrogen). The reactions were incubated in Simpliamp (Applied Biosystems) in a 96-well plate according to the manufacturer's instructions. Quantitative PCR (qPCR) was performed using FastStart Essential DNA Probes Master (Roche). The reactions were incubated in a LightCycler® 96 System (Roche) in a 96-well plate at 95°C for 10 min, followed by 40 cycles of 95°C for 15 sec and 60°C for 1 min. The threshold cycle (Ct) was defined as the fractional cycle number at which the fluorescence passed the fixed threshold. The amount of mRNA was normalized against that of GAPDH mRNA. RT primers, PCR primers, and probes were purchased as TaqMan™ Gene expression Assays from ThermoFisher Scientific (*GAPDH*, Hs02758991; *TNNT2*, Hs00943911; *ACTN2*, Hs00153809; *MYL2*, Hs00166405; *MYL7*, Hs01085598; *TNNI3*, Hs0016957; *GJA1*, Hs00748445; *EOMES*, Hs00172872; *MIXL1*, Hs00430824; *MESP1*, Hs00251489; *NANOG*, Hs02387400; *OCT4*, Hs04260367; *SOX2*, and Hs04234836). In the TaqMan hPSC Scorecard Panel assay, according to the manufacturer's protocol, the cDNA was synthesized using the High-Capacity cDNA Reverse Transcription Kit with RNase Inhibitor (Applied Biosystems); PCR was performed using 2 × TaqMan Fast Advanced Master Mix (Applied Biosystems) and the reactions were incubated in StepOnePlus Real-Time PCR System (Applied Biosystems) in a 96-well plate. Analysis was performed by inputting data into the hPSC Scorecard analysis software (Thermo Fisher Scientific).

miRNA microarray analysis

Total RNA including miRNA was isolated from cell culture supernatant using a miRNeasy Serum/Plasma Kit (Qiagen, Valencia, CA). RNA quantity and quality were determined using an Agilent 2100 Bioanalyzer

(Agilent Technologies, Inc.). RNA was labeled with cyanine 3 (Cy3) using miRNA Complete Labeling and Hyb Kit (Agilent Technologies, Inc.) following the manufacturer’s instructions. Briefly, RNA was dephosphorylated using Calf Intestinal Alkaline Phosphatase (CIP) Master Mix incubated at 37°C for 30 min. Dephosphorylated RNA was denatured with DMSO incubated at 100°C for 5 min and then immediately transferred to ice for 2 min. These products were mixed with a Ligation Master Mix for T4 RNA Ligase and Cy3-pCp (Cyanine 3-Cytidine biphosphate) and incubated at 16°C for 2 h. Labeled RNA was dried using a vacuum concentrator at 55°C for 1.5 h. Cy3-pCp-labeled RNA was hybridized on Agilent Human miRNA Microarray (Design ID: 070156) array at 55°C for 20 h. After washing, microarrays were scanned using an Agilent SureScan Microarray Scanner System (G4900DA). Intensity values for each scanned feature were quantified using Agilent Feature Extraction software version 12.1.1.1, which performs background subtractions. To identify miRNAs that are useful for monitoring mesoderm/cardiac differentiation and maturation processes, we evaluated the expression ratio of each miRNA at two specific time points. For the residual undifferentiated markers and the mesoderm/cardiac differentiation markers, we selected those with high expression ratios in the respective appropriate pair of samples and considered any reported significance as markers in the literature, if available. As criteria for selecting cardiac maturation markers, we considered not only a higher expression ratio in day 51 samples compared to day 9, day 21, and day 35 samples but also a consistent trend of either remaining constant or increasing monotonically over time.

Microarray analysis

Total RNA was isolated from cell culture supernatant using a ReliaPrep™ RNA Cell Miniprep System (Promega) according to the manufacturer’s instructions. Total RNA quantity and quality were determined using NanoDrop One spectrophotometer (Thermo Fisher Scientific Inc.) and Agilent 2100 Bioanalyzer (Agilent Technologies, Santa Clara, CA). For cRNA amplification and labeling, Agilent Low Input Quick Amp Labeling Kit (Agilent Technologies) was used following the manufacturer’s instructions. Briefly, total RNA was reverse-transcribed to double-strand cDNA using poly dT-T7 promoter primer at 65℃ for 10 min, then incubated at 40℃ for 2 h with 5X First Strand Buffer, 0.1 M DTT, 10 mM dNTP mix, and Affinity Script Rnase Block Mix. The Affinity Script enzyme was inactivated at 70℃ for 15 min. The cDNA products were used as templates for in vitro transcription to generate fluorescent cRNA. cDNA template were combined with 5x Transcription Buffer, 0.1 M DTT, NTP mix, T7 RNA polymerase, and Cyanine3-CTP, followed by incubation at 40℃ for 2 h. Labeled cRNAs were purified using Rneasy mini spin columns (Qiagen) and eluted in 30 μl

of nuclease-free water. The cRNA quantity and Cyanine-3 (Cy3) dye incorporation were determined using NanoDrop One spectrophotometer and Agilent 2100 Bioanalyzer. For each hybridization, 0.60 µg of Cy3 dye-labeled cRNA were fragmented and hybridized at 65℃ for 17 h using the Agilent SurePrint G3 Human GE v3 8x60K Microarray (Design ID: 072363). After washing, the microarrays were scanned using Agilent SureScan Microarray Scanner (G4900DA). Intensity values of each scanned feature were quantified using Agilent Feature Extraction software version 12.1.1.1, which performs background subtractions. We only used features that were flagged as no errors (Detected flags) and excluded features that were not positive, significant, uniform, above background, and saturated, and population outliers (Not Detected and Compromised flags). Quantile Normalization was performed using Agilent GeneSpring software version 14.9.1.

Calcium transients

To examine intracellular calcium transients, hiPSC-CMs were incubated in 1mM Cal 520, AM (AAT Bioquest, 21130) for 90 min at 37°C and analyzed with a BZ-X710 microscope and BZ-X800 Analyzer (Keyence) 30 min after room temperature incubation. The time to 50% decay was calculated from the decreasing limbs of the transients.

Statistical analysis

All statistical analysis was performed using Prism (GraphPad). All data are expressed as mean ± SD. All differences between the two group analyses were determined with the paired t-test. $P < 0.05$ was considered statistically significant.

REFERENCES

Kameda, K., Someya, S., Fujita, J., Fukuda, K., and Tohyama, S. (2022). Protocol for enhanced proliferation of human pluripotent stem cells in tryptophan-fortified media. STAR Protoc *3*, 101341. 10.1016/j.xpro.2022.101341.

Someya, S., Tohyama, S., Kameda, K., Tanosaki, S., Morita, Y., Sasaki, K., Kang, M.I., Kishino, Y., Okada, M., Tani, H., et al. (2021). Tryptophan Metabolism Regulates Proliferative Capacity of Human Pluripotent Stem Cells. iScience *24*, 102090. 10.1016/j.isci.2021.102090.

Tano, K., Yasuda, S., Kuroda, T., Saito, H., Umezawa, A., and Sato, Y. (2014). A novel in vitro method for detecting undifferentiated human pluripotent stem cells as impurities in cell therapy products using a highly efficient culture system. PLoS One *9*, e110496. 10.1371/journal.pone.0110496.

Tanosaki, S., Akiyama, T., Kanaami, S., Fujita, J., Ko, M.S.H., Fukuda, K., and Tohyama, S. (2022). Purification of cardiomyocytes and neurons derived from human pluripotent stem cells by inhibition of de novo fatty acid synthesis. STAR Protoc *3*, 101360. 10.1016/j.xpro.2022.101360.

Tohyama, S., Fujita, J., Fujita, C., Yamaguchi, M., Kanaami, S., Ohno, R., Sakamoto, K., Kodama, M., Kurokawa, J., Kanazawa, H., et al. (2017). Efficient Large-Scale 2D Culture System for Human Induced Pluripotent Stem Cells and Differentiated Cardiomyocytes. Stem Cell Reports *9*, 1406-1414. 10.1016/j.stemcr.2017.08.025.

Tohyama, S., Fujita, J., Hishiki, T., Matsuura, T., Hattori, F., Ohno, R., Kanazawa, H., Seki, T., Nakajima, K., Kishino, Y., et al. (2016). Glutamine Oxidation Is Indispensable for Survival of Human Pluripotent Stem Cells. Cell Metab *23*, 663-674. 10.1016/j.cmet.2016.03.001.

Tohyama, S., Hattori, F., Sano, M., Hishiki, T., Nagahata, Y., Matsuura, T., Hashimoto, H., Suzuki, T., Yamashita, H., Satoh, Y., et al. (2013). Distinct metabolic flow enables large-scale purification of mouse and human pluripotent stem cell-derived cardiomyocytes. Cell Stem Cell *12*, 127-137. 10.1016/j.stem.2012.09.013.

Yoshida, S., Kato, T.M., Sato, Y., Umekage, M., Ichisaka, T., Tsukahara, M., Takasu, N., and Yamanaka, S. (2023). A clinical-grade HLA haplobank of human induced pluripotent stem cells matching approximately 40% of the Japanese population. Med (N Y) *4*, 51-66.e10. 10.1016/j.medj.2022.10.003.
