# Supplementary material for: Seamless and non-destructive monitoring of extracellular microRNAs during cardiac differentiation from human pluripotent stem cells
Source: Stem Cell Reports. 2023 Sep 21;18(10):1925–39. doi: 10.1016/j.stemcr.2023.08.011 (PMC10656301; doi:10.1016/j.stemcr.2023.08.011)
Supplement: Document S2. Article plus supplemental information [file mmc2.pdf]

Stem Cell Reports
Article
OPEN ACCESS

Seamless and non-destructive monitoring of extracellular microRNAs during cardiac differentiation from human pluripotent stem cells

Otoya Sekine,[1] Sayaka Kanaami,[1,3] Kanako Masumoto,[4] Yuki Aihara,[4] Yuika Morita-Umei,[1,5] Hidenori Tani,[1,6] Yusuke Soma,[1] Tomohiko C. Umei,[1] Kotaro Haga,[1] Taijun Moriwaki,[1] Yujiro Kawai,[2] Masatoshi Ohno,[2] Yoshikazu Kishino,[1] Hideaki Kanazawa,[1] Keiichi Fukuda,[1,3] Masaki Ieda,[1] and Shugo Tohyama[1,*]
[1]Department of Cardiology, Keio University School of Medicine, 35 Shinanomachi, Shinjuku-ku, Tokyo 160-8582, Japan
[2]Department of Cardiovascular Surgery, Keio University School of Medicine, 35 Shinanomachi, Shinjuku-ku, Tokyo 160-8582, Japan
[3]Heartseed Inc, The Artcomplex Center of Tokyo, #302, 12-9, Daikyo-cho, Shinjuku-ku, Tokyo 160-0015, Japan
[4]Sysmex Corporation, Central Research Laboratories, 4-4-4 Takatsukadai, Nishi-ku, Kobe 651-2271, Japan
[5]Kanagawa Institute of Industrial Science and Technology (KISTEC), Kawasaki, Kanagawa, Japan
[6]Joint Research Laboratory for Medical Innovation in Heart Disease, Keio University School of Medicine, 35 Shinanomachi, Shinjuku-ku, Tokyo 160-8582, Japan
*Correspondence: shugotohyama@keio.jp
https://doi.org/10.1016/j.stemcr.2023.08.011

SUMMARY

Monitoring cardiac differentiation and maturation from human pluripotent stem cells (hPSCs) and detecting residual undifferentiated hPSCs are indispensable for the development of cardiac regenerative therapy. MicroRNA (miRNA) is secreted from cells into the extracellular space, and its role as a biomarker is attracting attention. Here, we performed an miRNA array analysis of supernatants during the process of cardiac differentiation and maturation from hPSCs. We demonstrated that the quantification of extracellular miR-489-3p and miR-1/133a-3p levels enabled the monitoring of mesoderm and cardiac differentiation, respectively, even in clinical-grade mass culture systems. Moreover, extracellular let-7c-5p levels showed the greatest increase with cardiac maturation during long-term culture. We also verified that residual undifferentiated hPSCs in hPSC-derived cardiomyocytes (hPSC-CMs) were detectable by measuring miR-302b-3p expression, with a detection sensitivity of 0.01%. Collectively, we demonstrate that our method of seamlessly monitoring specific miRNAs secreted into the supernatant is non-destructive and effective for the quality evaluation of hPSC-CMs.

INTRODUCTION

Severe heart failure is a common ailment globally. Although several drug- and device-based therapies are being developed, currently, the only treatment that can fundamentally improve severely reduced heart function is heart transplantation, because the impaired heart does not self-regenerate. Unfortunately, because heart transplantation cannot become a standard treatment because of donor shortages, alternative treatments are required (Khush et al., 2018). Cardiac regenerative therapy using human pluripotent stem cells (hPSCs) is a novel technique that is currently attracting attention (Kawaguchi et al., 2021; Shiba et al., 2016; Takahashi et al., 2007) and rapidly advancing into clinical realization. However, hundreds of millions of cells are needed to treat a single patient with heart failure. Thus, developing a quality evaluation method for cardiomyocytes (CMs) derived from hPSCs is indispensable. Particularly, detecting residual undifferentiated hPSCs, which may cause teratoma after transplantation, is vital (Miura et al., 2009; Sekine et al., 2020). In addition, the differentiation of a large number of hPSC-derived CMs (hPSC-CMs) required for transplantation is expensive, and the cardiac differentiation efficiency is inconsistent, despite recent advances in the differentiation protocols. Hence, a method for monitoring the mesoderm and cardiac differentiation efficiency is also required to avoid unproductive large-scale culture with wasted costs and efforts (Morita et al., 2022). Moreover, because the maturity of hPSC-CMs affects the engraftment rate and arrhythmia after transplantation (Funakoshi et al., 2016; Wu et al., 2021), methods for evaluating the maturity of hPSC-CMs for transplantation are also required. However, generally, in the evaluation of the quality of final products, some cells are destroyed, indicating that the cells that will actually be transplanted cannot be evaluated directly, and many costly produced cells are required for quality evaluation. Therefore, the ideal scenario is to develop a non-destructive method for quality evaluation during mesoderm/cardiac differentiation and maturation from hPSCs.

MicroRNAs (miRNAs) are non-coding single-stranded ribonucleotides containing about 22 nucleotides that can bind to mRNA at the poly-adenylated tail end (3′-UTR) in the non-coding region to inhibit or promote its degradation (Ambros, 2004; He and Hannon, 2004; Huntzinger and Izaurralde, 2011). miRNAs are secreted from cells bound to specific proteins or encapsulated in exosomes (Turchinovich et al., 2011), and their biological function and role as a biomarker are being investigated (Creemers et al., 2012; Zhou et al., 2016). Many studies have shown that detecting miRNAs secreted in body fluids can be used as a diagnostic and prognostic tool for several diseases

This is an open access article under the CC BY license (http://creativecommons.org/licenses/by/4.0/).

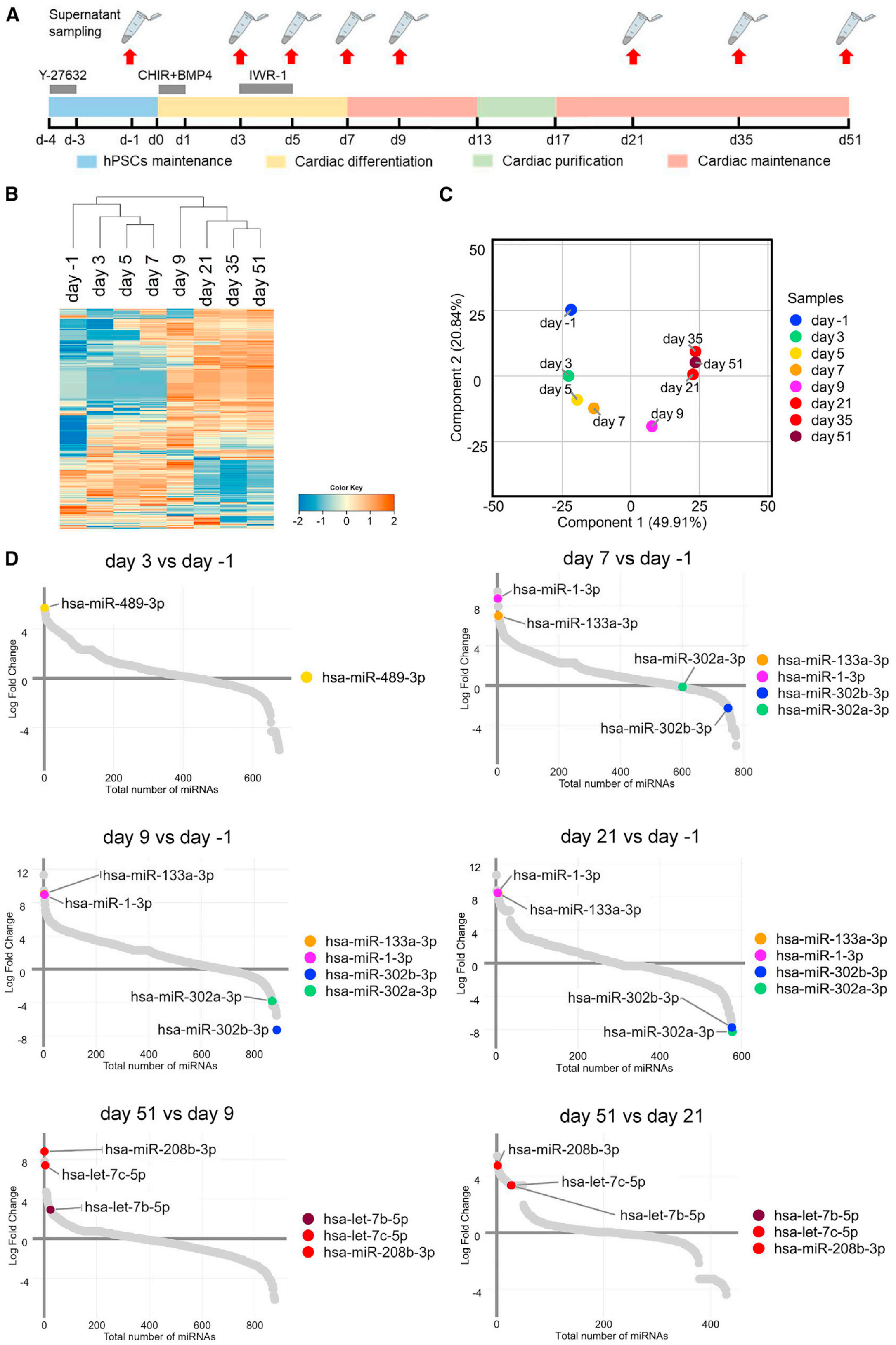

(legend on next page)

(Schwarzenbach et al., 2011; Vegter et al., 2016; Wang et al., 2011). As for their biological function, some miRNAs secreted from hPSC-derivatives or cardiac progenitor cells improve impaired cardiac function (Jung et al., 2017; Wang et al., 2019). We previously developed an efficient method for extracting miRNAs from the supernatant and accurately quantifying them (Masumoto et al., 2022) and hypothesized that quantifying the miRNA in the culture supernatant could be a useful, non-destructive method for quality evaluation during mesoderm/cardiac differentiation and maturation from hPSCs.

In this study, we investigated which miRNAs were the most suitable for the detection of residual undifferentiated hPSCs and monitoring mesoderm/cardiac differentiation and maturation, and eventually identified undifferentiated human induced PSC (hiPSC)-, mesodermal cell-, immature CM-, and matured CM-specific secreted miRNAs via a miRNA array analysis using supernatant samples during the mesoderm/cardiac differentiation and maturation process from hiPSCs. We also applied the findings to a clinical-grade hiPSC-CMs production system for regenerative therapy.

RESULTS

miRNA array analysis of the supernatant during cardiac differentiation and maturation

First, we performed an miRNA array analysis to analyze the miRNAs secreted into the supernatant during the mesoderm/cardiac differentiation and maturation process from hiPSCs. Supernatant samples were collected on day −1, day 3, day 5, day 7, day 9, day 21, day 35, and day 51 (Figure 1A). We identified 925 miRNAs secreted in these supernatants, most of which overlapped among samples (Figure 1B). Principal component analysis showed that the miRNA profiles secreted into the supernatant changed dramatically during the mesoderm/cardiac differentiation and maturation process (Figure 1C). To identify the miRNAs useful for monitoring the mesoderm/cardiac differentiation and maturation process, the ratio of the expression of each miRNA at two specific time points was assessed. The miRNAs with a high ratio of day 3 to day −1 were used as markers for mesoderm differentiation, those with a high ratio of day 7 (or day 9) to day −1 were used as markers for cardiac differentiation, those with a high ratio of day 51 to day 9 (or day 21, day35) were used as markers for cardiac maturation, and those with a high ratio of day −1 to day 9 (or day 21) were used as markers for residual undifferentiated hiPSCs in hiPSC-CMs. From these data, we extracted the candidate miRNAs for each stage. Among those miRNAs with the highest ratios at the various stages, we selected miR-489-3p as a marker for mesoderm differentiation; miR-1-3p and miR-133a-3p as markers for cardiac differentiation; miR-208b-3p, let-7b-5p, and let-7c-5p as markers for cardiac maturation; and miR-302a-3p and miR-302b-3p as markers for residual undifferentiated hPSCs (Figure 1D). We then performed real-time RT-qPCR analyses for these miRNAs, confirmed by following the Minimum Information for Publication of Quantitative Real-Time PCR Experiment guidelines (Bustin et al., 2009). The linear dynamic changes between copy numbers and cycle threshold (Ct) values were observed from 1×10^3 to 1×10^7 copies for synthetic miR-489-3p, miR-1-3p, miR-133a-3p, miR-208b-3p, and let-7c-5p and from 500–10^7 copies for synthetic miR-302b-3p and synthetic miR-302a-3p (Figure S1). Within this range, the measured Ct values were converted into absolute copy numbers using a standard curve generated from synthetic miRNAs.

Monitoring of mesoderm and cardiac differentiation

To investigate whether the expressions of miR-489-3p, miR-1-3p, and miR-133a-3p change according to the differentiation efficiency, we measured the levels of miRNAs secreted in the supernatant during mesoderm and cardiac differentiation from hiPSCs using RT-qPCR. For efficient cardiac differentiation, we sequentially supplemented GSK3β inhibitor (CHIR99021), bone morphogenic protein (BMP) 4, and Wnt inhibitor (IWR1) to RPMI medium plus B27 without insulin. Subsequently, to control the differentiation efficiency, we induced CMs with or without CHIR99021, BMP4, and IWR1 and eventually divided the cells into three groups: high differentiation efficiency (group H), intermediate differentiation efficiency (group I), and low differentiation efficiency (group L) (Figure 2A). To confirm mesoderm differentiation, immunofluorescence staining for Brachyury T, MIXL1, and EOMES was performed after differentiation. The results showed that almost all cells were positive for Brachyury T, MIXL1, and EOMES in groups H and I (H/I), whereas no positive cells

Figure 1. The profile of miRNAs secreted into the supernatant during cardiac differentiation verified by miRNA array analysis

(A) Differentiation protocol for cardiac differentiation from hiPSCs (253G4) and the timing of supernatant collection are shown.

(B) Heatmap showing the changes in expression levels of all miRNAs detected in the supernatant during cardiac differentiation.

(C) Principal component analysis showing stepwise changes in the profile of miRNAs secreted into the supernatant during cardiac differentiation.

(D) The ratio of each miRNA's expression in the supernatant between two specific time points arranged in descending order. miRNAs with particularly high ratios are selected.

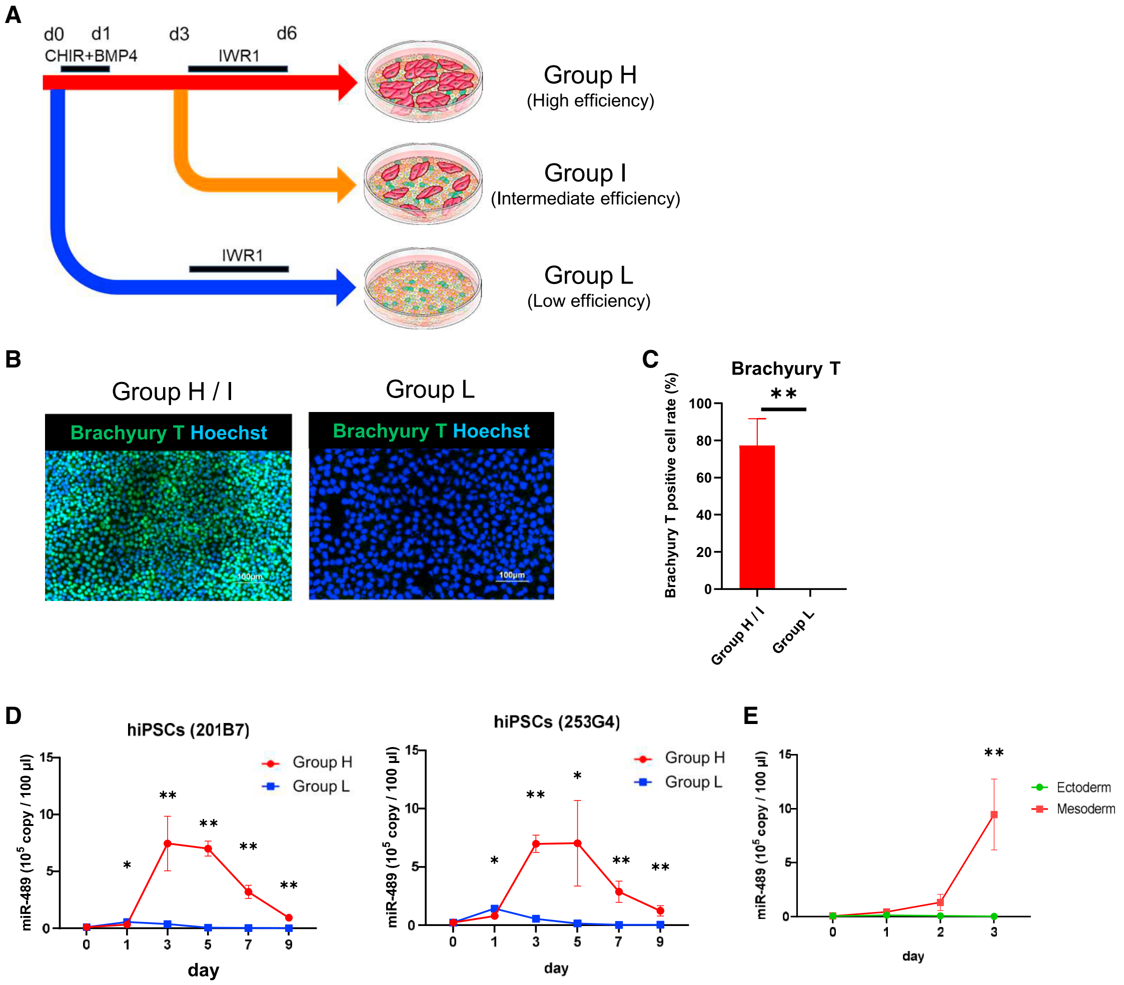

Figure 2. Changes in the levels of miR-489-3p secreted into the culture supernatant during mesoderm and cardiac differentiation

(A) Mesoderm and cardiac differentiation protocol induced with or without CHIR99021, BMP4, and IWR1, and divided into three groups: high differentiation efficiency (group H), intermediate differentiation efficiency (group I), and low differentiation efficiency (group L).

(B) Immunostaining for Brachyury T (green) and Hoechst (blue) in group H/I and L on day 1 differentiated from hiPSCs (253G4). Scale bar, 100 μm.

(C) Mesoderm differentiation efficiency described by measuring the Brachyury T-positive cell proportion (n = 3 independent experiments for 253G4 cell lines).

(D) miR-489-3p levels in the culture supernatant during cardiac differentiation in groups H and L (n = 3 independent experiments for both the 201B7 and 253G4 cell lines).

(E) miR-489-3p levels in the culture supernatant during neural and cardiac differentiation from hiPSCs (n = 3 independent experiments for 253G4 cell lines). Data are presented as mean ± SD; *p < 0.05; **p < 0.01. All p values are determined with a ratio-paired t test.

were detected in group L (Figures 2B and S2A). The mesoderm differentiation efficiency, i.e., the proportion of Brachyury T-positive cells in immunofluorescence-stained images for hiPSCs (253G4), was 77.3% ± 14.4% in group H/I and 0% in group L (Figure 2C). We also performed RT-qPCR to detect the expression levels of *MESP1*, *EOMES*, and *MIXL1* on day 3 and observed high expression of these markers in group H/I but no or poor expression in group L (Figure S2B). Moreover, because CD13 is known as one of cardiac mesoderm markers (Skelton et al., 2016), we evaluated CD13-positive cells using flow cytometry and observed a high proportion of CD13-positive cells in group H/I (63.4% ± 0.5%), whereas few cells were positive in group L (0.9% ± 0.3%) on day 3 after differentiation (Figures S2C and S2D). We also examined the tri-lineage differentiation ability and pluripotent marker gene expression of the two groups on day 3 using Scorecard assays, which generate an algorithmic score based on the expression of 96 genes analyzed via qPCR (Bock et al., 2011). Group H/I showed higher mesoderm scores and lower

scores for pluripotency. In contrast, group L showed lower mesoderm scores and higher ectoderm scores (Figures S2E and S2F). These results indicate that group H/I was well differentiated predominantly in the mesoderm lineage and group L showed the tendency of ectoderm differentiation. Notably, miR-489-3p expression level in the supernatant during the differentiation process increased sharply on day 3 in group H, whereas there was no increase in group L (Figure 2D). Based on the data of Scorecard assays in group L, we also performed neural differentiation from hiPSCs as a representative of ectodermal differentiation and confirmed that increased miR-489-3p expression was not observed during neural differentiation (Figures 2E and S2G). In addition, for other mesoderm cell lineages, miR-489-3p expression was significantly increased during endothelial cell differentiation from hiPSCs on day 3 after differentiation (Figures S2H and S2I). We also assessed intracellular miR-489-3p expression during cardiac differentiation from hiPSCs and observed a marked increase on day 3 (Figure S2J). These results indicate that miR-489-3p is useful for monitoring mesoderm differentiation.

In addition, to confirm cardiac differentiation in groups H, I, and L, cardiac differentiation efficiency was determined by measuring the proportion of cardiac troponin T-positive cells via flow cytometry on day 10 after differentiation. The percentage positivity was 94.0% ± 1.4% (201B7) and 86.8% ± 10.5% (253G4) in group H, 23.1% ± 20.7% (201B7) and 40.8% ± 25.0% (253G4) in group I, and 0.2% ± 0.1% (201B7) and 0.1 ± 0.2% (253G4) in group L (Figures 3B and 3C). The proportion of cardiac MLC2a-positive cells, determined via flow cytometry, tended to be generally consistent with that of cTnT-positive cells for each group on day 10 (Figures S3B and S3C). In addition, immunofluorescent staining for several cardiac markers (cTnT, α-actinin, MLC2a, and cTnI) showed that the percentage of cardiac marker-positive cells was significantly higher in group H than in group I, and almost non-existent in group L on day 10 after differentiation (Figures 3A and S3A). We performed RT-qPCR to detect the expression levels of *TNNT2*, *ACTN2*, and *MYL7* and observed high expression of these markers in group H, moderate expression in group I, and poor expression in group L (Figure S3D). The RT-qPCR data showed that the expression level of miR-1-3p in the supernatant during cardiac differentiation transiently increased on day 7, then dramatically decreased on day 8. In contrast, miR-133a-3p expression in the supernatant increased continuously from days 7–8. The expression levels of both miRNAs were significantly different between group H/I after day 7 (Figure 3D). Intracellular miR-1-3p and miR-133a-3p expression levels were also evaluated and showed the same trend as in the supernatant (Figure S3E). The coefficient of determination for the correlation between the cardiac differentiation efficiency and the expression levels of miR-1-3p and miR-133a-3p in the supernatant on day 7 were 0.7061 and 0.8018, respectively, for 201B7 hiPSCs, and 0.5731 and 0.4815, respectively, for 253G4 hiPSCs (Figure S3F). These results indicate that measuring extracellular miR-1-3p and miR-133a-3p levels is useful for monitoring cardiac differentiation efficiency.

To verify the adaptability in three-dimensional (3D) culture, we evaluated the expression levels of miR-1-3p and miR-133a-3p in the supernatant in the 3D culture system using a bioreactor and observed a sharp increase in their levels on day 7 (Figure S4A). Immunostaining of multiple cell aliquots from the same bioreactor for α-actinin and vimentin showed large variations in the cardiac differentiation efficiency for each embryoid body (Figures S4B–S4D). These data suggest that assessing differentiation efficiency using a subset of cells within a 3D culture system can result in variability. In contrast, our method using miRNAs in the supernatant, which reflects the whole cell population, holds potential for application in 3D culture systems.

Application of the monitoring system in a clinical-grade mass culture system

To apply our findings to a clinical-grade mass culture system, we induced CMs from the human leukocyte antigen homozygous hiPSC line QHJI14s04 for clinical use using animal-free and chemically defined culture medium with large four-layer culture plates and investigated whether high or low cardiac differentiation efficiency could be determined by measuring the levels of miR-1-3p and miR-133a-3p in the supernatant (Figure 4A). In this experiment, sampling of supernatants was performed during the standard cardiac differentiation protocol each time (Figure 4A), the proportion of cTnT-positive cells was measured via flow cytometry, and the batches were divided into high (>90%; 94.8% ± 3.0%) and intermediate differentiation efficiency groups (<65%; 49.1% ± 13.3%) (Figures 4B and 4C). The expression levels of miR-1-3p and miR-133a-3p on day 7 were significantly different between the two groups (Figure 4D). The coefficients of determination for the correlation between the cardiac differentiation efficiency and the expression levels of miR-1-3p and miR-133a-3p in the supernatant on day 7 were 0.7466 and 0.7216, respectively, suggesting that high and intermediate efficiency batches can be distinguished nondestructively at an early stage in cardiac differentiation (Figure 4E).

Monitoring of cardiac maturation

Long-term culture of hiPSC-CMs promotes maturation (Karbassi et al., 2020; Tani and Tohyama, 2022; Wu et al., 2021). Therefore, we performed immunostaining and RT-qPCR for maturation-related markers and

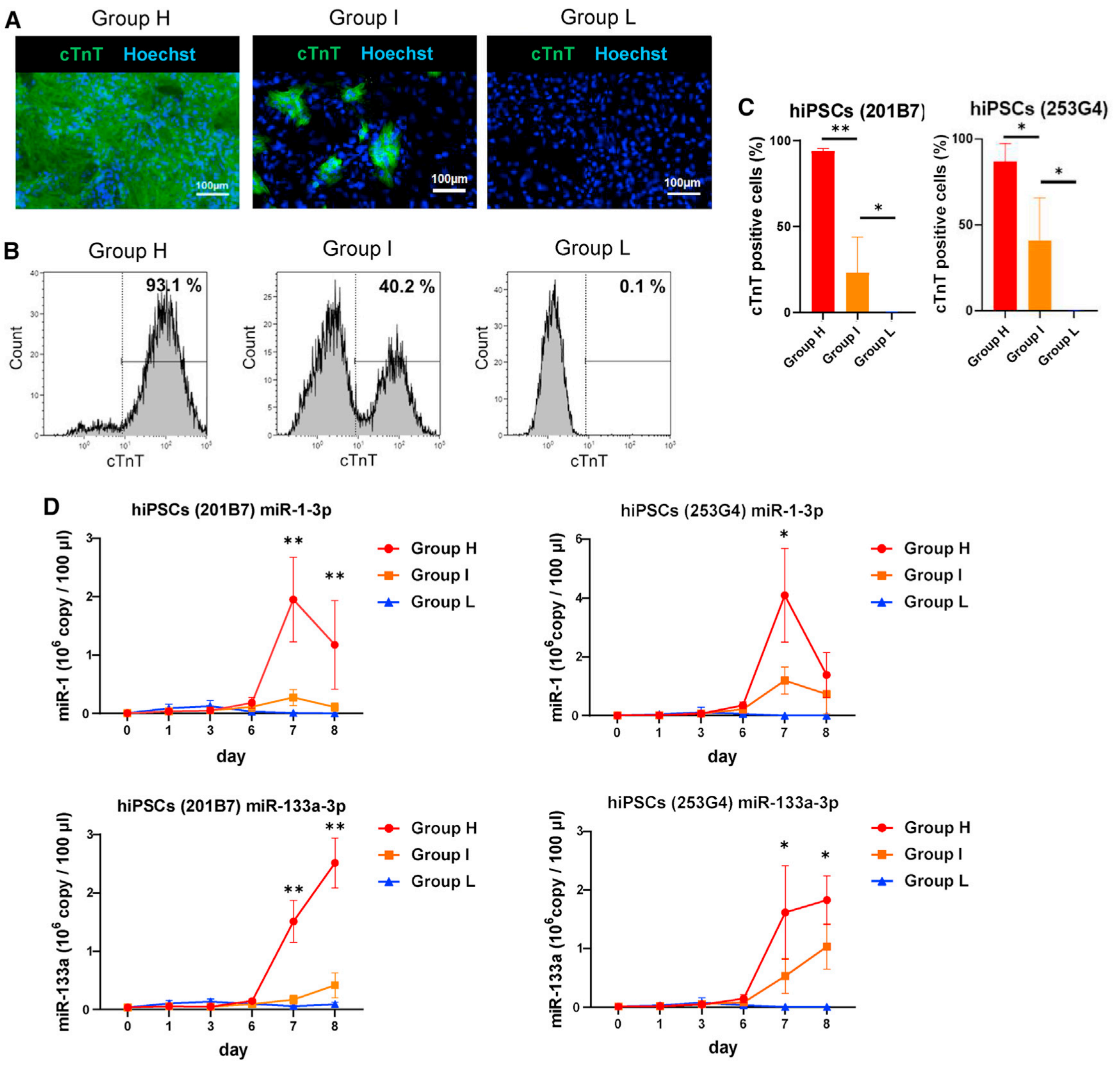

Figure 3. Changes in the levels of miR-1-3p, and miR-133a-3p secreted into the culture supernatant during cardiac differentiation
(A) Immunostaining for cardiac troponin T (cTnT) (green) and Hoechst (blue) in groups H, I, and L on day 10 differentiated from hiPSCs (253G4). Scale bar, 100 μm.
(B and C) Flow cytometry analysis for cTnT-positive cells in groups H, I, and L on day 10 (n = 6 independent experiments for the 201B7 cell line and n = 4 independent experiments for the 253G4 cell line).
(D) miR-1-3p and miR-133a-3p levels in the culture supernatant during cardiac differentiation for groups H, I, and L (n = 6 independent experiments for the 201B7 cell line and n = 4 independent experiments for the 253G4 cell line). Statistical analysis was performed between group H and I. Data are presented as mean ± SD; *p < 0.05; **p < 0.01. All p values are determined with a ratio-paired t test.

confirmed that hiPSC-CMs displayed mature profiles after long-term culture in cardiac maintenance medium (low-glucose medium [MEMα] plus 5% fetal bovine serum [FBS]) (Figures 5A, 5C, and S5A). Immunostaining for N-cadherin showed a marked increase in cell area during the long-term culture process from days 20–50 (Figure 5B). To comprehensively evaluate the changes in gene expressions during long-term culture, transcriptome analysis was performed on hiPSC-CMs on days 20, 35, and 50, and increased expression of multiple cardiac maturation markers was observed (Figure 5D). Moreover, to confirm physiological maturation during long-term culture, Ca transient was examined in hiPSC-CMs on days 20 and 50. We demonstrated that long-term culture increased the peak amplitude of fluorescence (F/F_0) and maximal upstroke velocity, prolonged 50% time to decay, and reduced the beating rate, suggesting physiological cardiac maturation (Figures 5E and 5F). Next, to identify maturation-related miRNAs in the supernatant, we performed RT-qPCR for let-7b-5p, let-7c-5p, and miR-208b-3p, which

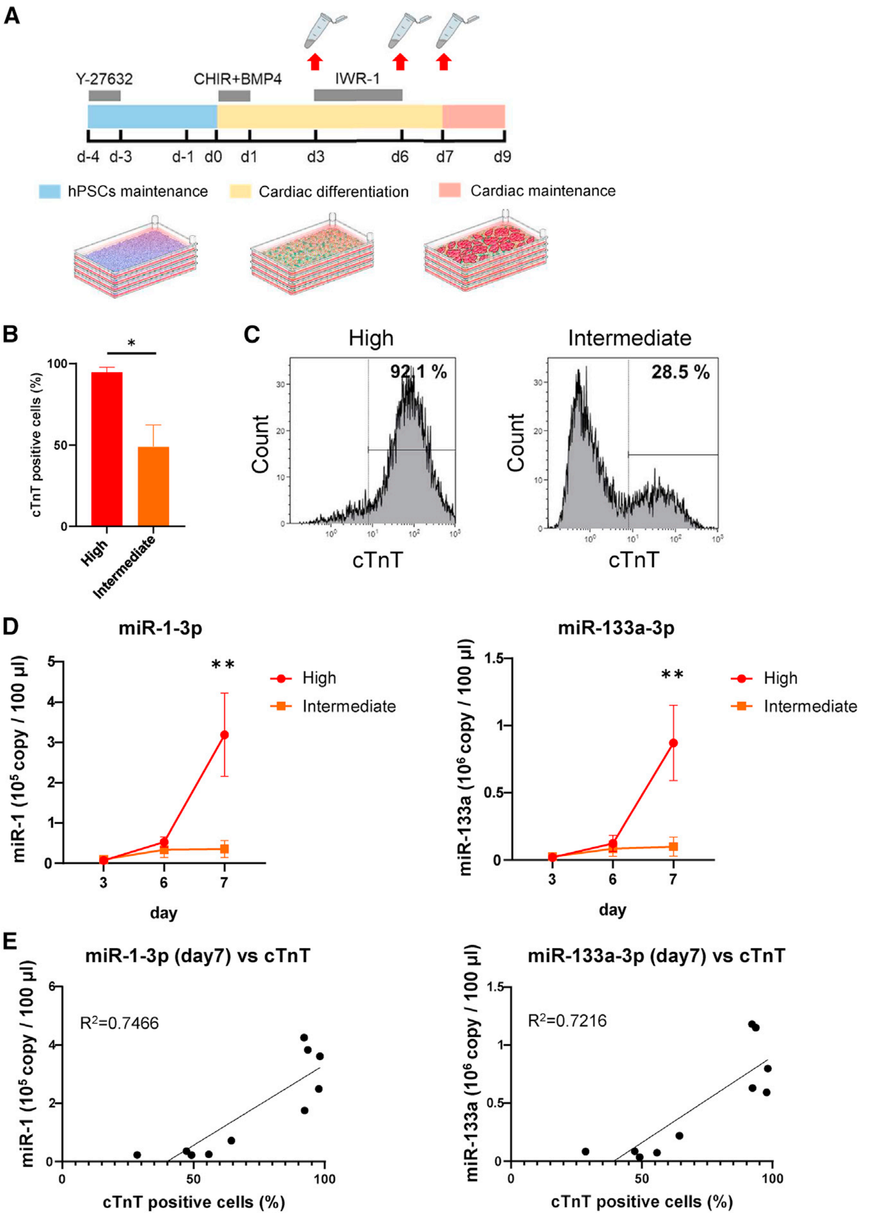

Figure 4. Application of cardiac differentiation monitoring by measuring miR-1-3p and miR-133a-3p levels in culture supernatant under clinical-grade large-scale culture

(A) Differentiation protocol for cardiac differentiation from hiPSCs in clinical-grade large-scale cultures and the timing of supernatant collection are shown.

(B and C) Flow cytometry analysis for cTnT-positive cells in two groups on day 10 (n = 5 independent experiments).

(D) miR-1-3p and miR-133a-3p levels in the culture supernatant during cardiac differentiation in groups High and Intermediate (n = 5 independent experiments).

(E) Correlation between the percentage of cTnT-positive cells on day 10 and the amount of miR-1-3p and miR-133a-3p in the culture supernatant on day 7. R^2, Coefficient of determination. Data are presented as mean ± SD; $^*p < 0.05$; $^{**}p < 0.01$. All p values are determined with a ratio-paired t test.

were listed as candidate molecules based on our miRNA array data. The results showed that the expression of let-7b-5p and miR-208b-3p in the supernatant did not significantly increase, whereas that of let-7c-5p markedly increased over time (Figures 5G, 5H, and S5B). We further assessed the expression of other let-7 family miRNAs and miR-499-5p, which is reported to be a CM-specific miRNA, in the supernatant and observed no remarkable increases in the expression of these markers over time compared with let-7c-5p (Figure S5B). The expression of intracellular let-7c-5p expression showed a remarkable increase during the mesoderm/cardiac differentiation and maturation (Figure S5C). We also evaluated the expression of miR-1-3p, miR-133a-3p, and let-7c-5p in the supernatant during the long-term culture of hiPSC-CMs. The expression of miR-1-3p in the supernatant increased during the cardiac differentiation phase, that of miR-133a-3p increased during the early cardiac maturation phase, and that of let-7c-5p increased during the late cardiac maturation phase (Figure 5G). Regarding the observation that miR-1-3p and miR-133a-3p expression temporarily decreases at approximately day 14 and then increases again, we confirmed that this pattern is also reflected in the intracellular expression levels with a transient decrease at approximately day 14 followed by a subsequent re-increase (Figure S5D).

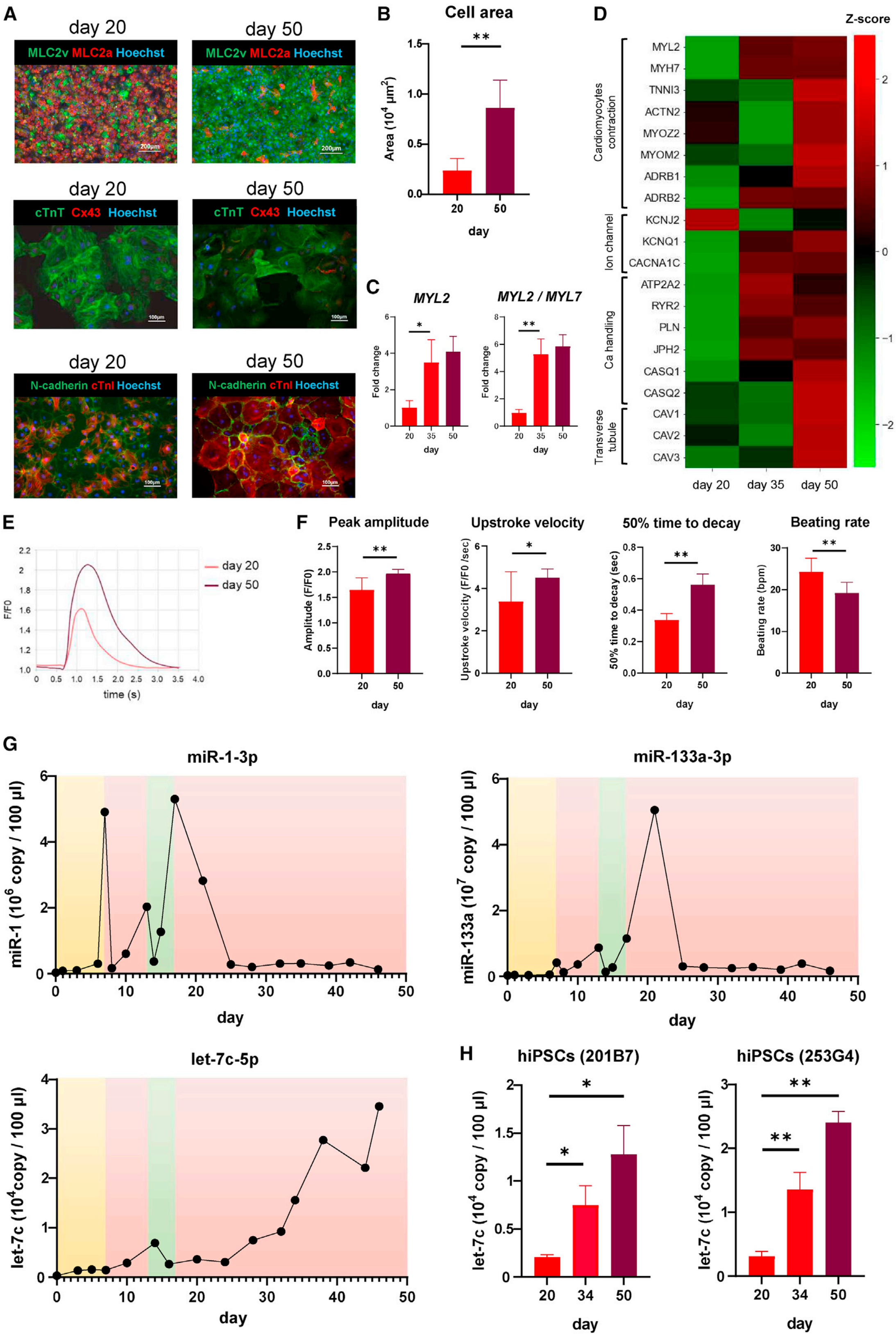

(legend on next page)

Detection of contaminating undifferentiated hiPSCs

The levels of miR-302a-3p and miR-302b-3p, which were listed as hiPSC-specific miRNAs based on the miRNA array data, were measured in the supernatant using RT-qPCR during mesoderm/cardiac differentiation and maturation. Extracellular miR-302a-3p and miR-302b-3p levels decreased during cardiac differentiation but a small amount of these miRNAs was detected in immature hiPSC-CMs at the cardiac differentiation phase; however, these levels were decreased in the long-term culture (Figure 6A). As less miR-302b-3p than miR-302a-3p was secreted from hiPSC-CMs, miR-302b-3p was considered a useful marker for the detection of residual undifferentiated hiPSCs in hiPSC-CMs (Figure 6A). Intracellular miR-302b-3p expression also showed a gradual decrease during the long-term culture (Figure S6A). First, we examined whether residual undifferentiated hiPSCs can be detected nondestructively using the miR-302b-3p expression in the supernatant after cardiac differentiation. On day 17 after cardiac differentiation, hiPSC-CMs, which were confirmed that the contamination of undifferentiated hiPSCs was fewer than 0.001% via colony formation assay (CFA), was seeded with hiPSCs at concentrations of 0%, 0.01%, and 0.1% on iMatrix-221-coated plates under cardiac maintenance conditions. The expression of miR-302b-3p in the supernatant was then measured by RT-qPCR (Figure 6B). miR-302b-3p was detected in the supernatant on day 20 even in hiPSC-CMs (both 201B7 and 253G4) cultures with 0% hiPSC contamination, whereas it decreased below the minimum limit of determination by day 28 (Figure 6D). In contrast, miR-302b-3p was consistently detected in the supernatant when hiPSC-CMs cultures (both 201B7 and 253G4) were contaminated with 0.01% or 0.1% hiPSCs (Figure 6D). Consistently, immunostaining on day 20 showed OCT4-and SSEA4-positive cells were not detected in hiPSC-CM cultures with 0% hiPSCs but were detected in hiPSC-CM cultures with 0.01% hiPSCs (Figure 6C). To compare our method with a conventional method, the expressions of hPSC-specific markers (*OCT4*, *NANOG*, and *SOX2*) were measured by RT-qPCR, and all of them were expressed in hiPSC-CMs on days 23–30 by more than 10^{-4} (0.01%) compared with those in undifferentiated hiPSCs (Figure S6B). We then investigated the feasibility of combining the CFA method with miR-302b-3p measurement in the supernatant by performing another experiment using hiPSC maintenance medium with iMatrix-511. On day 17, hiPSC-CMs were seeded with hiPSCs at concentrations of 0% and 0.001% on iMatrix-511-coated plates, and the levels of miR-302b-3p in the supernatant were measured and compared between the groups (Figure 6E). Unlike cultures using cardiac maintenance medium, when hiPSC maintenance medium was used, the secretion of miR-302b-3p from hiPSC-CM cultures containing 0% hiPSCs did not fall below the minimum limit of determination, even on day 30. The expression of miR-302b-3p on day 20 was similar in both groups, but the amount of secretion gradually increased in the hiPSC-CM cultures with 0.001% hiPSCs, and there was a significant difference from approximately day 26 (Figure 6G). Immunostaining showed that OCT4- and SSEA4-positive cells were not detected in hiPSC-CM cultures with 0% hiPSCs, whereas large OCT4-and SSEA4-positive colonies were detected in the hiPSC-CM cultures with 0.001% hiPSCs on day 28 (Figure 6F). We evaluated intracellular miR-302b-3p expression from hiPSC-CMs containing 0% hiPSCs under both cardiac and hiPSC maintenance medium. Unlike its extracellular expression, intracellular miR-302b-3p expression consistently decreased not only under cardiac maintenance conditions, but also under hiPSC maintenance medium during the culture process (Figure S6C).

Figure 5. miRNAs that significantly increase in the supernatant during cardiac maturation

(A) Immunostaining for MLC2v (green) with MLC2a (red), cTnT (green) with Cx43 (red), and N-cadherin (green) with cTnI (red) in hiPSC (253G4)-CMs cultures on days 20 and 50. Scale bar, 200 μm for MLC2v and MLC2a and 100 μm for others.

(B) The cell area measured in the immunostaining image for N-cadherin in hiPSC (253G4)-CMs cultures on days 20 and 50 (n = 30 cells in three independent cultures for 253G4 cell lines).

(C) The expression of *MYL2* and the ratio of *MYL2* to *MYL7* in the long-term cultured hiPSC-CMs on days 20, 35, and 50, as measured by RT-qPCR (n = 4 independent experiments for 253G4 cell lines).

(D) Microarray analysis showing the profile of transcripts expressed in the long-term cultured hiPSC (253G4)-CMs on days 20, 35, and 50. Heatmap showing the changes in the transcriptional expression levels of several cardiac maturation markers detected in each sample.

(E) Representative Ca transients of hiPSC (253G4)-CMs cultures on days 20 and 50.

(F) Peak of amplitude magnitudes, maximal upstroke velocity, 50% time to decay, and beating rates measured in the Ca transients of hiPSC (253G4)-CMs cultures on days 20 and 50 (n = 12 independent experiments).

(G) miR-1-3p, miR-133a-3p, and let-7c-5p levels in the culture supernatant during cardiac differentiation and long-term culture of hiPSC (253G4)-CMs.

(H) let-7c-5p levels in the culture supernatant during long-term culture of hiPSC-CMs on days 20, 34, and 50 (n = 3 independent experiments for both the 201B7 and 253G4 cell lines). Data are presented as mean ± SD; *p < 0.05; **p < 0.01. All p values are determined with a ratio-paired t test.

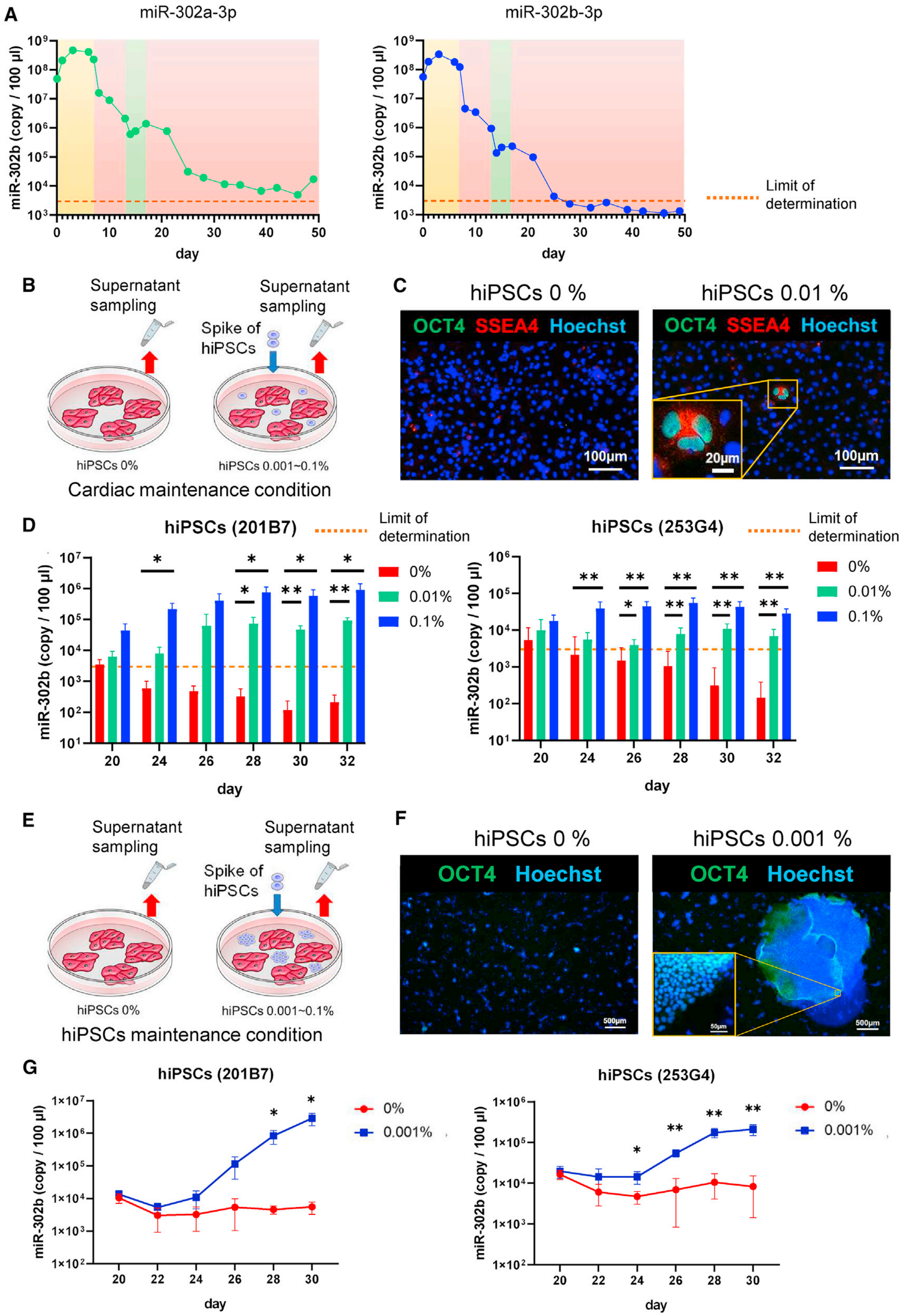

(legend on next page)

DISCUSSION

In this study, we demonstrated that the various miRNAs secreted from hiPSC-derivatives into culture supernatants change during the mesoderm/cardiac differentiation and maturation process and we identified stage-specific miRNAs useful for monitoring mesoderm/cardiac differentiation and maturation, and detection of residual undifferentiated hPSCs. The quantification of extracellular miRNAs enabled us to evaluate the quality of large numbers of hPSC derivatives for transplantation without destroying them. In addition, our miRNA extraction method has higher extraction efficiency and less variation than the conventional method, leading to more accurate quantification of the copy numbers of miRNAs in supernatants (Masumoto et al., 2022). A technique has been reported that visualizes intracellular miRNA expression and monitors differentiation using miRNA-responsive non-viral reporter vectors (Nakanishi et al., 2017). However, the authors mentioned that their approach carries a risk of insertional mutagenicity and is not suitable for clinical applications, which require strict low mutagenicity risk. We believe that our method is a practical non-invasive monitoring approach suitable for clinical use, considering its safety and technical simplicity.

It has been reported that the secretion of miRNA into extracellular space is actively regulated and differs partially from the intracellular expression profile (Pigati et al., 2010). Although some studies have reported that miRNA secreted into extracellular space is abundant in vesicles, such as exosomes (Valadi et al., 2007), other studies showed that the proportion of miRNA that is secreted by binding to proteins and without being capsulated in vesicles is even higher (Zhou et al., 2016). In this study, all miRNAs present in the supernatant were extracted for both the miRNA array analysis and RT-qPCR.

The miR-302 family has been reported to be expressed in hPSCs (Lakshmipathy et al., 2010). Although small amounts of miR-302b are secreted from hPSC-CMs in the early differentiation period, residual undifferentiated hPSCs with a contamination rate of over 0.01% can be detected in the culture supernatant 28 days after the induction of cardiac differentiation. Several methods for detecting residual undifferentiated hPSCs have been reported (Sekine et al., 2020; Wang et al., 2020); however, many of these require the destruction of cells from the final products, indicating that the final products are not evaluated as a whole. In other words, the possibility that undifferentiated hPSCs remain in parts other than the collected sample cannot be ruled out. Thus, a non-destructive and holistic evaluation method is required. A method using a recombinant peptide corresponding with the N-terminal domain of the BC2L-C protein has been reported as a non-destructive method for detecting undifferentiated hPSCs from culture supernatant (Tateno et al., 2014). In contrast, our method demonstrated the capability to detect as few as 0.01% hiPSCs in hiPSC-CMs. Compared with previously reported non-invasive detection methods, our method exhibits exceptional sensitivity in detecting residual undifferentiated hPSC, even in the presence of nonspecific expression resulting from a large number of hiPSC-CMs. The CFA, i.e., detecting undifferentiated hPSCs by culturing a sample of transplant cells in hPSC maintenance culture conditions, has been previously reported. In this assay, the detection limit for undifferentiated hPSCs contamination was 0.01%–0.001% (Tano et al., 2014). However, because contaminating hPSCs are detected by immunostaining, the CFA is non-quantitative and has a risk of overlooking hPSC contamination. In the present study, by combining the CFA with the measurement of miR-302b-3p levels in the culture supernatant, it was possible to quantitatively detect residual undifferentiated hPSCs with 0.001% sensitivity.

miR-489-3p, which was discovered as a marker of mesoderm differentiation, has been reported to be an onco-suppressor miRNA that negatively regulates cell proliferation

Figure 6. Verification of the usefulness of miR-302b-3p for the detection of residual undifferentiated hiPSCs in hiPSC-CM cultures

(A) miR-302a-3p and miR-302b-3p levels in the culture supernatant during cardiac differentiation and long-term culture of hiPSC (253G4)-CMs.

(B) Schematic representation of spike-in of hiPSCs with hiPSC-CM cultures and supernatant sampling in cardiac maintenance conditions.

(C) Immunostaining for OCT4 (green), SSEA4 (red), and Hoechst (blue) in cultures of hiPSC-CMs with 0% or 0.01% spiked-in hiPSCs (253G4) in cardiac maintenance medium on day 20. Scale bar, 100 μm.

(D) miR-302b-3p levels in the culture supernatant during culture of hiPSC-CMs with each concentration of hiPSCs in cardiac maintenance medium (n = 3 independent experiments for both the 201B7 and 253G4 cell lines).

(E) Schematic representation of spike-in of hiPSCs with hiPSC-CM cultures and supernatant sampling in hiPSC maintenance conditions.

(F) Immunostaining for OCT4 (green) and Hoechst (blue) in cultures of hiPSC-CMs with 0% or 0.001% spiked-in hiPSCs (253G4) in hiPSC maintenance medium on day 28.

(G) miR-302b-3p levels in the culture supernatant during culture of hiPSC-CMs with each concentration of hiPSCs in hiPSC maintenance medium (n = 3 independent experiments for both the 201B7 and 253G4 cell lines). Data are presented as mean ± SD; *$p < 0.05$; **$p < 0.01$. All p values are determined with a ratio-paired t test.

(Li et al., 2017; Zhang et al., 2016), suggesting that it may also be involved in the decline of the cell cycle during mesoderm differentiation from hPSCs. In the present study, we demonstrated a rapid increase in the extracellular secretion of miR-489-3p on day 3 of the mesoderm differentiation process not only in the case of cardiac differentiation but also in endothelial cell differentiation, representing other mesodermal lineages. Notably, this increase was not present when mesoderm differentiation was not induced or during active induction of ectoderm differentiation, such as neural differentiation.

miR-1 and miR-133a have been reported to be highly expressed in CMs (Lagos-Quintana et al., 2002); it is also reported that expression of miR-1 and miR-133a is increased during the early cardiac differentiation process and that miR-1 and miR-133a themselves negatively regulate cardiac differentiation and proliferation (Cordes and Srivastava, 2009; Liu and Olson, 2010; Zhao et al., 2005). Our study showed that extracellular secretion of miR-1-3p and miR-133a-3p increased rapidly on day 7 during cardiac differentiation from hPSCs, with a good correlation with cardiac differentiation efficiency. Moreover, miR-133a has been reported to be involved in the transition from glycolysis to oxidative phosphorylation (Hua et al., 2021), which is consistent with our previous data, which showed a switch from glycolysis to oxidative phosphorylation during cardiac differentiation (Tohyama et al., 2016), and may be useful as a marker of cardiac differentiation and early cardiac maturation.

Hundreds of millions of hPSC-CMs are required for cardiac regenerative therapy for humans. During the manufacturing process for large numbers of hPSC-CMs, the ability to evaluate the cardiac differentiation efficiency via supernatant sampling in the early phase is useful because it can prevent wasted costs and efforts. Particularly, in a two-dimensional (2D) mass culture system, it is necessary to detach all cells from plates to collect some cells. In contrast, the supernatant can be collected non-invasively at any time point during differentiation, and the evaluation target is the whole cell population, which is advantageous. Therefore, we applied our method to clinical-grade mass culture systems for CM production using HLA homozygous hiPSC lines for clinical use. In a 3D culture system, it is possible to collect a portion of the cells for evaluation at any time point without detaching all cells from the plates as in 2D culture, but the disadvantage remains that it is limited to the evaluation of only small part of cells for transplantation. We showed that cardiac differentiation efficiency differs among aliquots of cells cultured in the same container and that the extracellular expression levels of miR-1-3p and miR-133a-3p in 3D culture systems undergo similar fluctuations as those in 2D culture. Therefore, we believe that the miRNA-based evaluation system using supernatants is a powerful tool not only in 2D culture systems, but also in 3D culture systems.

A previous report showed that intracellular expression of let-7 family members increases in hPSC-CMs during long-term culture and the let-7 family itself accelerates cardiac maturation (Kuppusamy et al., 2015). The let-7 family is reportedly suppressed by *LIN28* and the LIN28–let-7 axis regulates the phosphatidylinositol 3-kinase-protein kinase B signaling pathway and the metabolic shift from cell proliferation and glycolysis to β oxidation during cardiac maturation (Ma et al., 2014). Our data showed that extracellular secretion of let-7c-5p is more significantly elevated than that for the other let-7 family members, suggesting that let-7c-5p may be a new marker for non-invasively assessing the maturity of hPSC-CMs.

There are some limitations to the present study that should be considered when interpreting the results. The levels of molecules secreted into the supernatant may change dependent on the cell density. Therefore, when applying this monitoring method to clinical manufacture, it will be necessary to create evaluation criteria specific for certain seeded cell density and culture conditions. In addition, a small amount of miR-302b-3p is secreted from immature hPSC-CMs, so it is desirable to use matured hPSC-CMs for undifferentiated hPSCs detection. We believe that there is room to consider whether miR-302b-3p, which is secreted in small quantities from the hPSC-CMs, can be used to evaluate the maturity of the hPSC-CMs. Although there are some challenges of its clinical applications, our method enabled seamless and non-destructive monitoring and quality evaluation of hiPSC-CMs. This technique also has the potential to be applied in various other organs and diseases after the identification of the relevant miRNAs.

EXPERIMENTAL PROCEDURES

Resource availability

Corresponding author

Further information and requests for resources and reagents should be directed to and will be fulfilled by the corresponding author, Shugo Tohyama (shugotohyama@keio.jp).

Materials availability

All data are available in the main text or the supplemental information. This study did not generate new unique reagents.

Data and code availability

The microarray data generated during this study have been deposited in the Gene Expression Omnibus (GEO). The accession numbers for the miRNA array and microarray data used in this study are GSE:240790 (miRNA array in supernatant samples) and GSE:240792 (microarray in hiPSC-CMs).

miRNA extraction and quantitative RT-PCR

For extraction from supernatant samples, miRNAs were isolated from 100 μL of supernatants using a modified Boom method (Boom et al., 1990) with magnetic beads (Sicastar-M, 39-00-153,

CoreFront). For extraction from cell samples, miRNAs were isolated from approximately 5 × 10^5–3 × 10^6 cells using a High Pure miRNA Isolation Kit (Roche). Of the 100 μL purified miRNA, 5–16 μL was reverse-transcribed into cDNA by MultiScribe reverse transcriptase (4311235, Applied Biosystems). The reactions were incubated in Simpliamp (Applied Biosystems) in a 96-well plate for 30 min at 16°C, 30 min at 42°C, 5 min at 85°C, and then held at 4°C. Real-time PCR was performed by Hot Start ExTaq PCR enzyme (RR006B, TaKaRa) using a standard TaqMan PCR protocol on LightCycler 96 System (Roche). The reactions were incubated in a 96-well plate at 95°C for 10 min, followed by 40 cycles of 95°C for 15 s and 60°C for 1 min. The Ct was defined as the fractional cycle number at which the fluorescence passed the fixed threshold. Ct values were converted into absolute copy numbers using a standard curve from synthetic miRNA. Stem-loop RT primers, PCR primers, and probes for miR-302a-3p and miR-302b-3p were designed as in previous papers (Chen et al., 2005; Masumoto et al., 2022). These primers and probes for miR-489-3p, miR-1-3p, miR-133a-3p, let-7a-5p, let-7b-5p, let-7c-5p, let-7d-5p, let-7e-5p, let-7f-5p, let-7g-5p, let-7i-5p, miR-208b-3p, miR-499a-5p, and RNU6B were purchased as a TaqMan MicroRNA Assay from ThermoFisher Scientific (miR-489-3p, 002358; miR-1-3p, 002222; miR-133a-3p, 002246; let-7a-5p, 000377; let-7b-5p, 002619; let-7c-5p, 000379; let-7d-5p, 002283; let-7e-5p, 002406; let-7f-5p, 000382; let-7g-5p, 002282; let-7i-5p, 002221; miR-208b-3p, 002290; miR-499a-5p, 001352; RNU6B, 001093). The evaluation of the intracellular amount of miRNA was normalized against that of RNU6B.

Preparation of samples containing diluted or spiked-in hiPSCs

Confluent hiPSCs (50%–70%) were dissociated using TrypLE (Thermo Fisher Scientific) for 3 min at room temperature. Dissociated cells were centrifuged and the cell number was counted by Vi-CELL XR (Beckman Coulter) by taking the average of three to five individual counts. Low-concentration solutions were achieved by serial dilution (maximal 9:1 ratio per dilution). On day 17, hiPSC-CMs were dissociated to single cells using L-Trypsin (Nacalai Tesque) at 37°C for 5 min. Populations of hiPSCs and hiPSC-CMs were combined to generate spiked samples containing 0.001 to 0.1% hiPSCs in 2–3 × 10^6 hiPSC-CMs and passaged them with hPSC maintenance medium supplemented with 10 μM Y-27632 into six-well plates coated with iMatrix-221 (Nippi, NP892-061) or iMatrix-511 (Nippi, NP892-011). After day 18, the cells were cultured in MEMα plus 5% FBS (cardiac maintenance medium) or hPSC maintenance medium, while the supernatant was collected, and the copy number of the miRNAs was measured by RT-qPCR. Immunostaining of the cocultured cells for OCT4 and SSEA4 was performed.

SUPPLEMENTAL INFORMATION

Supplemental information can be found online at https://doi.org/10.1016/j.stemcr.2023.08.011.

ACKNOWLEDGMENTS

The authors thank Tomoko Haruna, Shuta Minagawa, Rei Ohno, Kuniko Momoi, Miho Yamaguchi, Yuki Yamamoto, Yui Narita, Noriko Kabasawa, and Naoko Matsumoto for technical assistance with cell preparation and culture (Department of Cardiology, Keio University). The authors also thank CiRA for providing the hiPSC lines (253G4, 201B7, and QHJI14s04). The authors thank Sysmex Corporation and DNA chip research Inc. for the gift of miRNA extraction-related reagents and the support of miRNA array data analysis, respectively. The present work was supported by a research grant from the Japan Agency for Medical Research and Development (AMED) (grant no. 23bm1123010 to S.T.), KISTEC (to S.T.), the Japanese Circulation Society (to S.T.), and research funding from Heartseed Inc.

AUTHOR CONTRIBUTIONS

S.T. conceptualized and designed the study; O.S. performed and analyzed most experiments; S.K., K.M., Y.A., K.H. and the other co-authors contributed to specific experiments; O.S. and S.T. wrote the original draft; S.T. wrote, reviewed, and edited the manuscript; S.T. and K.F. acquired funding; S.T. and K.F. supervised the study.

DECLARATION OF INTERESTS

O.S., S.K., K.M., Y.A., K.F., and S.T. have a patent pending related to this work. K.F. is a co-founder and CEO of Heartseed, Inc. S.T. is an advisor to Heartseed Inc. S.K. is an employee of Heartseed Inc. M.K. and Y.A. are employees of Sysmex Corporation. S.T., H.K., and K.F. own equity in Heartseed, Inc. The remaining authors have no conflicts of interest to disclose.

Received: January 31, 2023
Revised: August 20, 2023
Accepted: August 21, 2023
Published: September 21, 2023

REFERENCES

Ambros, V. (2004). The functions of animal microRNAs. Nature *431*, 350–355. https://doi.org/10.1038/nature02871.

Bock, C., Kiskinis, E., Verstappen, G., Gu, H., Boulting, G., Smith, Z.D., Ziller, M., Croft, G.F., Amoroso, M.W., Oakley, D.H., et al. (2011). Reference Maps of human ES and iPS cell variation enable high-throughput characterization of pluripotent cell lines. Cell *144*, 439–452. https://doi.org/10.1016/j.cell.2010.12.032.

Boom, R., Sol, C.J., Salimans, M.M., Jansen, C.L., Wertheim-van Dillen, P.M., and van der Noordaa, J. (1990). Rapid and simple method for purification of nucleic acids. J. Clin. Microbiol. *28*, 495–503. https://doi.org/10.1128/jcm.28.3.495-503.1990.

Bustin, S.A., Benes, V., Garson, J.A., Hellemans, J., Huggett, J., Kubista, M., Mueller, R., Nolan, T., Pfaffl, M.W., Shipley, G.L., et al. (2009). The MIQE guidelines: minimum information for publication of quantitative real-time PCR experiments. Clin. Chem. *55*, 611–622. https://doi.org/10.1373/clinchem.2008.112797.

Chen, C., Ridzon, D.A., Broomer, A.J., Zhou, Z., Lee, D.H., Nguyen, J.T., Barbisin, M., Xu, N.L., Mahuvakar, V.R., Andersen, M.R., et al. (2005). Real-time quantification of microRNAs by stem-loop RT-PCR. Nucleic Acids Res. *33*, e179. https://doi.org/10.1093/nar/gni178.

Cordes, K.R., and Srivastava, D. (2009). MicroRNA regulation of cardiovascular development. Circ. Res. *104*, 724–732. https://doi.org/10.1161/circresaha.108.192872.

Creemers, E.E., Tijsen, A.J., and Pinto, Y.M. (2012). Circulating microRNAs: novel biomarkers and extracellular communicators in cardiovascular disease? Circ. Res. *110*, 483–495. https://doi.org/10.1161/circresaha.111.247452.

Funakoshi, S., Miki, K., Takaki, T., Okubo, C., Hatani, T., Chonabayashi, K., Nishikawa, M., Takei, I., Oishi, A., Narita, M., et al. (2016). Enhanced engraftment, proliferation, and therapeutic potential in heart using optimized human iPSC-derived cardiomyocytes. Sci. Rep. *6*, 19111. https://doi.org/10.1038/srep19111.

He, L., and Hannon, G.J. (2004). MicroRNAs: small RNAs with a big role in gene regulation. Nat. Rev. Genet. *5*, 522–531. https://doi.org/10.1038/nrg1379.

Hua, Y.T., Xu, W.X., Li, H., and Xia, M. (2021). Emerging roles of MiR-133a in human cancers. J. Cancer *12*, 198–206. https://doi.org/10.7150/jca.48769.

Huntzinger, E., and Izaurralde, E. (2011). Gene silencing by microRNAs: contributions of translational repression and mRNA decay. Nat. Rev. Genet. *12*, 99–110. https://doi.org/10.1038/nrg2936.

Jung, J.H., Fu, X., and Yang, P.C. (2017). Exosomes Generated From iPSC-Derivatives: New Direction for Stem Cell Therapy in Human Heart Diseases. Circ. Res. *120*, 407–417. https://doi.org/10.1161/circresaha.116.309307.

Karbassi, E., Fenix, A., Marchiano, S., Muraoka, N., Nakamura, K., Yang, X., and Murry, C.E. (2020). Cardiomyocyte maturation: advances in knowledge and implications for regenerative medicine. Nat. Rev. Cardiol. *17*, 341–359. https://doi.org/10.1038/s41569-019-0331-x.

Kawaguchi, S., Soma, Y., Nakajima, K., Kanazawa, H., Tohyama, S., Tabei, R., Hirano, A., Handa, N., Yamada, Y., Okuda, S., et al. (2021). Intramyocardial Transplantation of Human iPS Cell-Derived Cardiac Spheroids Improves Cardiac Function in Heart Failure Animals. JACC. Basic Transl. Sci. *6*, 239–254. https://doi.org/10.1016/j.jacbts.2020.11.017.

Khush, K.K., Cherikh, W.S., Chambers, D.C., Goldfarb, S., Hayes, D., Jr., Kucheryavaya, A.Y., Levvey, B.J., Meiser, B., Rossano, J.W., and Stehlik, J.; International Society for Heart and Lung Transplantation (2018). The International Thoracic Organ Transplant Registry of the International Society for Heart and Lung Transplantation: Thirty-fifth Adult Heart Transplantation Report-2018; Focus Theme: Multiorgan Transplantation. J. Heart Lung Transplant. *37*, 1155–1168. https://doi.org/10.1016/j.healun.2018.07.022.

Kuppusamy, K.T., Jones, D.C., Sperber, H., Madan, A., Fischer, K.A., Rodriguez, M.L., Pabon, L., Zhu, W.Z., Tulloch, N.L., Yang, X., et al. (2015). Let-7 family of microRNA is required for maturation and adult-like metabolism in stem cell-derived cardiomyocytes. Proc. Natl. Acad. Sci. USA *112*, E2785–E2794. https://doi.org/10.1073/pnas.1424042112.

Lagos-Quintana, M., Rauhut, R., Yalcin, A., Meyer, J., Lendeckel, W., and Tuschl, T. (2002). Identification of tissue-specific microRNAs from mouse. Curr. Biol. *12*, 735–739. https://doi.org/10.1016/s0960-9822(02)00809-6.

Lakshmipathy, U., Davila, J., and Hart, R.P. (2010). miRNA in pluripotent stem cells. Regen. Med. *5*, 545–555. https://doi.org/10.2217/rme.10.34.

Li, Y., Ma, X., Wang, Y., and Li, G. (2017). miR-489 inhibits proliferation, cell cycle progression and induces apoptosis of glioma cells via targeting SPIN1-mediated PI3K/AKT pathway. Biomed. Pharmacother. *93*, 435–443. https://doi.org/10.1016/j.biopha.2017.06.058.

Liu, N., and Olson, E.N. (2010). MicroRNA regulatory networks in cardiovascular development. Dev. Cell *18*, 510–525. https://doi.org/10.1016/j.devcel.2010.03.010.

Ma, X., Li, C., Sun, L., Huang, D., Li, T., He, X., Wu, G., Yang, Z., Zhong, X., Song, L., et al. (2014). Lin28/let-7 axis regulates aerobic glycolysis and cancer progression via PDK1. Nat. Commun. *5*, 5212. https://doi.org/10.1038/ncomms6212.

Masumoto, K., Aihara, Y., Miyagawa Kuroishi, M., Maeda, N., Sakai, Y., Oka, Y., Takahashi, Y., Oda, K., and Yanagida, M. (2022). Highly sensitive and non-disruptive detection of residual undifferentiated cells by measuring miRNAs in culture supernatant. Sci. Rep. *12*, 10351. https://doi.org/10.1038/s41598-022-14273-z.

Miura, K., Okada, Y., Aoi, T., Okada, A., Takahashi, K., Okita, K., Nakagawa, M., Koyanagi, M., Tanabe, K., Ohnuki, M., et al. (2009). Variation in the safety of induced pluripotent stem cell lines. Nat. Biotechnol. *27*, 743–745. https://doi.org/10.1038/nbt.1554.

Morita, Y., Kishino, Y., Fukuda, K., and Tohyama, S. (2022). Scalable manufacturing of clinical-grade differentiated cardiomyocytes derived from human-induced pluripotent stem cells for regenerative therapy. Cell Prolif. *55*, e13248. https://doi.org/10.1111/cpr.13248.

Nakanishi, H., Miki, K., Komatsu, K.R., Umeda, M., Mochizuki, M., Inagaki, A., Yoshida, Y., and Saito, H. (2017). Monitoring and visualizing microRNA dynamics during live cell differentiation using microRNA-responsive non-viral reporter vectors. Biomaterials *128*, 121–135. https://doi.org/10.1016/j.biomaterials.2017.02.033.

Pigati, L., Yaddanapudi, S.C.S., Iyengar, R., Kim, D.J., Hearn, S.A., Danforth, D., Hastings, M.L., and Duelli, D.M. (2010). Selective release of microRNA species from normal and malignant mammary epithelial cells. PLoS One *5*, e13515. https://doi.org/10.1371/journal.pone.0013515.

Schwarzenbach, H., Hoon, D.S.B., and Pantel, K. (2011). Cell-free nucleic acids as biomarkers in cancer patients. Nat. Rev. Cancer *11*, 426–437. https://doi.org/10.1038/nrc3066.

Sekine, K., Tsuzuki, S., Yasui, R., Kobayashi, T., Ikeda, K., Hamada, Y., Kanai, E., Camp, J.G., Treutlein, B., Ueno, Y., et al. (2020). Robust detection of undifferentiated iPSC among differentiated cells. Sci. Rep. *10*, 10293. https://doi.org/10.1038/s41598-020-66845-6.

Shiba, Y., Gomibuchi, T., Seto, T., Wada, Y., Ichimura, H., Tanaka, Y., Ogasawara, T., Okada, K., Shiba, N., Sakamoto, K., et al. (2016). Allogeneic transplantation of iPS cell-derived cardiomyocytes regenerates primate hearts. Nature *538*, 388–391. https://doi.org/10.1038/nature19815.

Skelton, R.J.P., Brady, B., Khoja, S., Sahoo, D., Engel, J., Arasaratnam, D., Saleh, K.K., Abilez, O.J., Zhao, P., Stanley, E.G., et al.

(2016). CD13 and ROR2 Permit Isolation of Highly Enriched Cardiac Mesoderm from Differentiating Human Embryonic Stem Cells. Stem Cell Rep. *6*, 95–108. https://doi.org/10.1016/j.stemcr.2015.11.006.

Takahashi, K., Tanabe, K., Ohnuki, M., Narita, M., Ichisaka, T., Tomoda, K., and Yamanaka, S. (2007). Induction of pluripotent stem cells from adult human fibroblasts by defined factors. Cell *131*, 861–872. https://doi.org/10.1016/j.cell.2007.11.019.

Tani, H., and Tohyama, S. (2022). Human Engineered Heart Tissue Models for Disease Modeling and Drug Discovery. Front. Cell Dev. Biol. *10*, 855763. https://doi.org/10.3389/fcell.2022.855763.

Tano, K., Yasuda, S., Kuroda, T., Saito, H., Umezawa, A., and Sato, Y. (2014). A novel *in vitro* method for detecting undifferentiated human pluripotent stem cells as impurities in cell therapy products using a highly efficient culture system. PLoS One *9*, e110496. https://doi.org/10.1371/journal.pone.0110496.

Tateno, H., Onuma, Y., Ito, Y., Hiemori, K., Aiki, Y., Shimizu, M., Higuchi, K., Fukuda, M., Warashina, M., Honda, S., et al. (2014). A medium hyperglycosylated podocalyxin enables noninvasive and quantitative detection of tumorigenic human pluripotent stem cells. Sci. Rep. *4*, 4069. https://doi.org/10.1038/srep04069.

Tohyama, S., Fujita, J., Hishiki, T., Matsuura, T., Hattori, F., Ohno, R., Kanazawa, H., Seki, T., Nakajima, K., Kishino, Y., et al. (2016). Glutamine Oxidation Is Indispensable for Survival of Human Pluripotent Stem Cells. Cell Metabol. *23*, 663–674. https://doi.org/10.1016/j.cmet.2016.03.001.

Turchinovich, A., Weiz, L., Langheinz, A., and Burwinkel, B. (2011). Characterization of extracellular circulating microRNA. Nucleic Acids Res. *39*, 7223–7233. https://doi.org/10.1093/nar/gkr254.

Valadi, H., Ekström, K., Bossios, A., Sjöstrand, M., Lee, J.J., and Lötvall, J.O. (2007). Exosome-mediated transfer of mRNAs and microRNAs is a novel mechanism of genetic exchange between cells. Nat. Cell Biol. *9*, 654–659. https://doi.org/10.1038/ncb1596.

Vegter, E.L., van der Meer, P., de Windt, L.J., Pinto, Y.M., and Voors, A.A. (2016). MicroRNAs in heart failure: from biomarker to target for therapy. Eur. J. Heart Fail. *18*, 457–468. https://doi.org/10.1002/ejhf.495.

Wang, L., Jia, Q., Xinnong, C., Xie, Y., Yang, Y., Zhang, A., Liu, R., Zhuo, Y., and Zhang, J. (2019). Role of cardiac progenitor cell-derived exosome-mediated microRNA-210 in cardiovascular disease. J. Cell Mol. Med. *23*, 7124–7131. https://doi.org/10.1111/jcmm.14562.

Wang, Z., Gagliardi, M., Mohamadi, R.M., Ahmed, S.U., Labib, M., Zhang, L., Popescu, S., Zhou, Y., Sargent, E.H., Keller, G.M., and Kelley, S.O. (2020). Ultrasensitive and rapid quantification of rare tumorigenic stem cells in hPSC-derived cardiomyocyte populations. Sci. Adv. *6*, eaay7629. https://doi.org/10.1126/sciadv.aay7629.

Wang, Z., Lu, Y., and Yang, B. (2011). MicroRNAs and atrial fibrillation: new fundamentals. Cardiovasc. Res. *89*, 710–721. https://doi.org/10.1093/cvr/cvq350.

Wu, P., Deng, G., Sai, X., Guo, H., Huang, H., and Zhu, P. (2021). Maturation strategies and limitations of induced pluripotent stem cell-derived cardiomyocytes. Biosci. Rep. *41*. BSR20200833. https://doi.org/10.1042/bsr20200833.

Zhang, B., Ji, S., Ma, F., Ma, Q., Lu, X., and Chen, X. (2016). miR-489 acts as a tumor suppressor in human gastric cancer by targeting PROX1. Am. J. Cancer Res. 6, 2021–2030.

Zhao, Y., Samal, E., and Srivastava, D. (2005). Serum response factor regulates a muscle-specific microRNA that targets Hand2 during cardiogenesis. Nature *436*, 214–220. https://doi.org/10.1038/nature03817.

Zhou, M., Hara, H., Dai, Y., Mou, L., Cooper, D.K.C., Wu, C., and Cai, Z. (2016). Circulating Organ-Specific MicroRNAs Serve as Biomarkers in Organ-Specific Diseases: Implications for Organ Allo- and Xeno-Transplantation. Int. J. Mol. Sci. *17*, 1232. https://doi.org/10.3390/ijms17081232.

Stem Cell Reports, Volume *18*

Supplemental Information

Seamless and non-destructive monitoring of extracellular microRNAs during cardiac differentiation from human pluripotent stem cells

Otoya Sekine, Sayaka Kanaami, Kanako Masumoto, Yuki Aihara, Yuika Morita-Umei, Hidenori Tani, Yusuke Soma, Tomohiko C. Umei, Kotaro Haga, Taijun Moriwaki, Yujiro Kawai, Masatoshi Ohno, Yoshikazu Kishino, Hideaki Kanazawa, Keiichi Fukuda, Masaki Ieda, and Shugo Tohyama

Figure S1

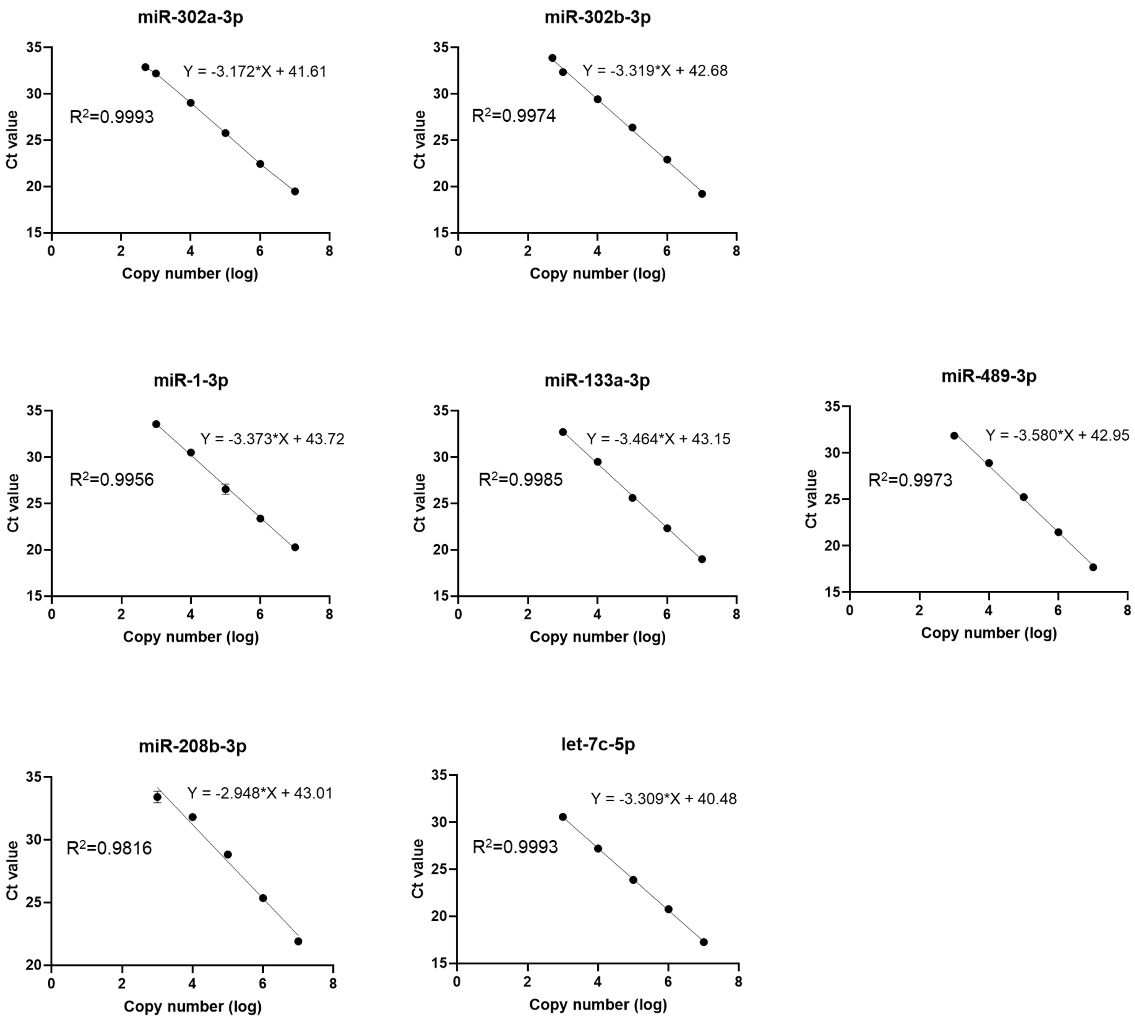

Figure S1. Correlation between the number of copies of each synthetic miRNA and the Ct value in RT-qPCR, related to Figure 1.

The linear dynamic changes between copy numbers and Ct values were 1×10^3–1×10^7 copies for synthetic miR-489-3p, miR-1-3p, miR-133a-3p, miR-208b-3p, and let-7c-5p and from 500–1×10^7 copies for synthetic miR-302a-3p and synthetic miR-302b-3p. R^2: Coefficient of determination.

Figure S2

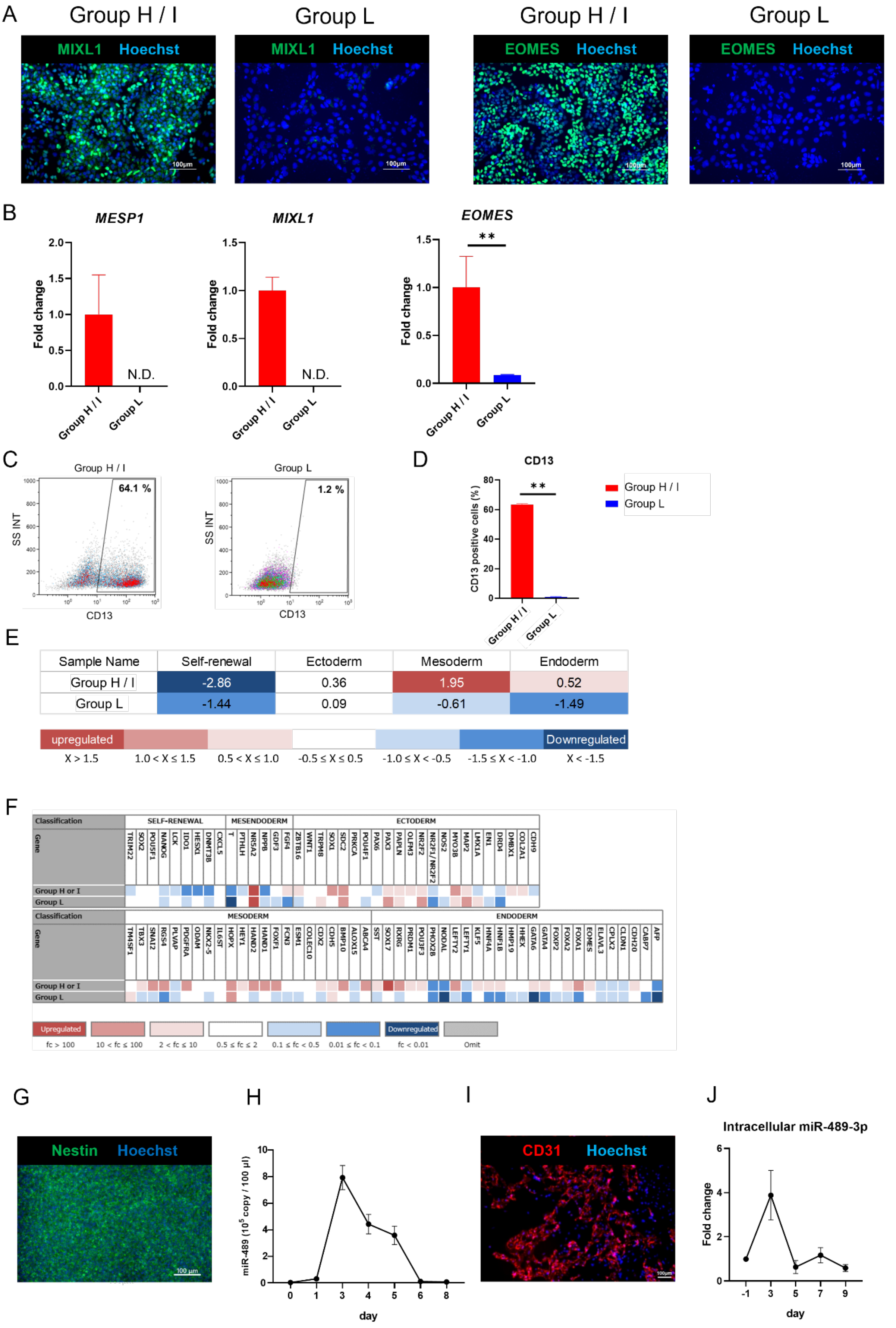
A
Group H / I
Group L
Group H / I
Group L
MIXL1 Hoechst
MIXL1 Hoechst
EOMES Hoechst
EOMES Hoechst
100μm
B
MESP1
MIXL1
EOMES
Fold change
N.D.
N.D.
**
Group H / I
Group L
C
Group H / I
Group L
64.1 %
1.2 %
SS INT
CD13
D
CD13
**
CD13 positive cells (%)
Group H / I
Group L
E
Sample Name | Self-renewal | Ectoderm | Mesoderm | Endoderm
Group H / I | -2.86 | 0.36 | 1.95 | 0.52
Group L | -1.44 | 0.09 | -0.61 | -1.49
upregulated
Downregulated
X > 1.5
1.0 < X ≤ 1.5
0.5 < X ≤ 1.0
-0.5 ≤ X ≤ 0.5
-1.0 ≤ X < -0.5
-1.5 ≤ X < -1.0
X < -1.5
F
Classification
Gene
SELF-RENEWAL
MESENDODERM
ECTODERM
MESODERM
ENDODERM
Group H or I
Group L
Upregulated
Downregulated
Omit
fc > 100
10 < fc ≤ 100
2 < fc ≤ 10
0.5 ≤ fc ≤ 2
0.1 ≤ fc < 0.5
0.01 ≤ fc < 0.1
fc < 0.01
G
Nestin Hoechst
100 μm
H
miR-489 (10^5 copy / 100 μl)
day
I
CD31 Hoechst
J
Intracellular miR-489-3p
Fold change
day

Figure S2. Verification of the usefulness of miR-489-3p for monitoring mesoderm differentiation, related to Figure 2.

(A) Immunostaining for MIXL1 or EOMES (green) and Hoechst (blue) in Groups H / I and L on day 3 differentiated from hiPSCs (253G4). Scale bar, 100 μm.

(B) RT-qPCR analysis of the relative expression levels of *MESP1*, *MIXL1*, and *EOMES* in Groups H / I and L on day 3 (n = 4 independent experiments for 253G4 cell lines).

(C and D) Flow cytometry analysis of CD13-positive cells in Groups H / I and L on day 3 (n = 3 independent experiments for the 253G4 cell line).

(E) Scorecard analysis for Group H / I and L on day 3 differentiated from hiPSCs (253G4). Algorithmic scores were generated by Scorecard analysis based on the expression of 96 genes per sample. Scores are a statistical comparison of the expression profile of the sample to that of the undifferentiated reference set.

(F) Heatmaps showing gene expression associated with trilineage differentiation and pluripotency in each sample. Colors correlate with the fold change in expression of the indicated gene relative to the undifferentiated reference set.

(G) Immunostaining for Nestin (green) and Hoechst (blue) in Neural stem cells on day 3 differentiated from hiPSCs (253G4). Scale bar, 100 μm.

(H) miR-489-3p levels in the culture supernatant during endothelial cell differentiation from hiPSCs (n = 3 independent experiments for 253G4 cell lines).

(I) Immunostaining for CD31 (red) and Hoechst (blue) in endothelial cells differentiated from hiPSCs (253G4) on day 8. Scale bar, 100 μm.

(J) miR-489-3p expression in the cells during mesoderm/cardiac differentiation from hiPSCs (n = 4 independent experiments for 253G4 cell lines).

Data are presented as mean ± SD; $*p < 0.05$; $**p < 0.01$. All p values are determined with a ratio-paired *t*-test.

Figure S3

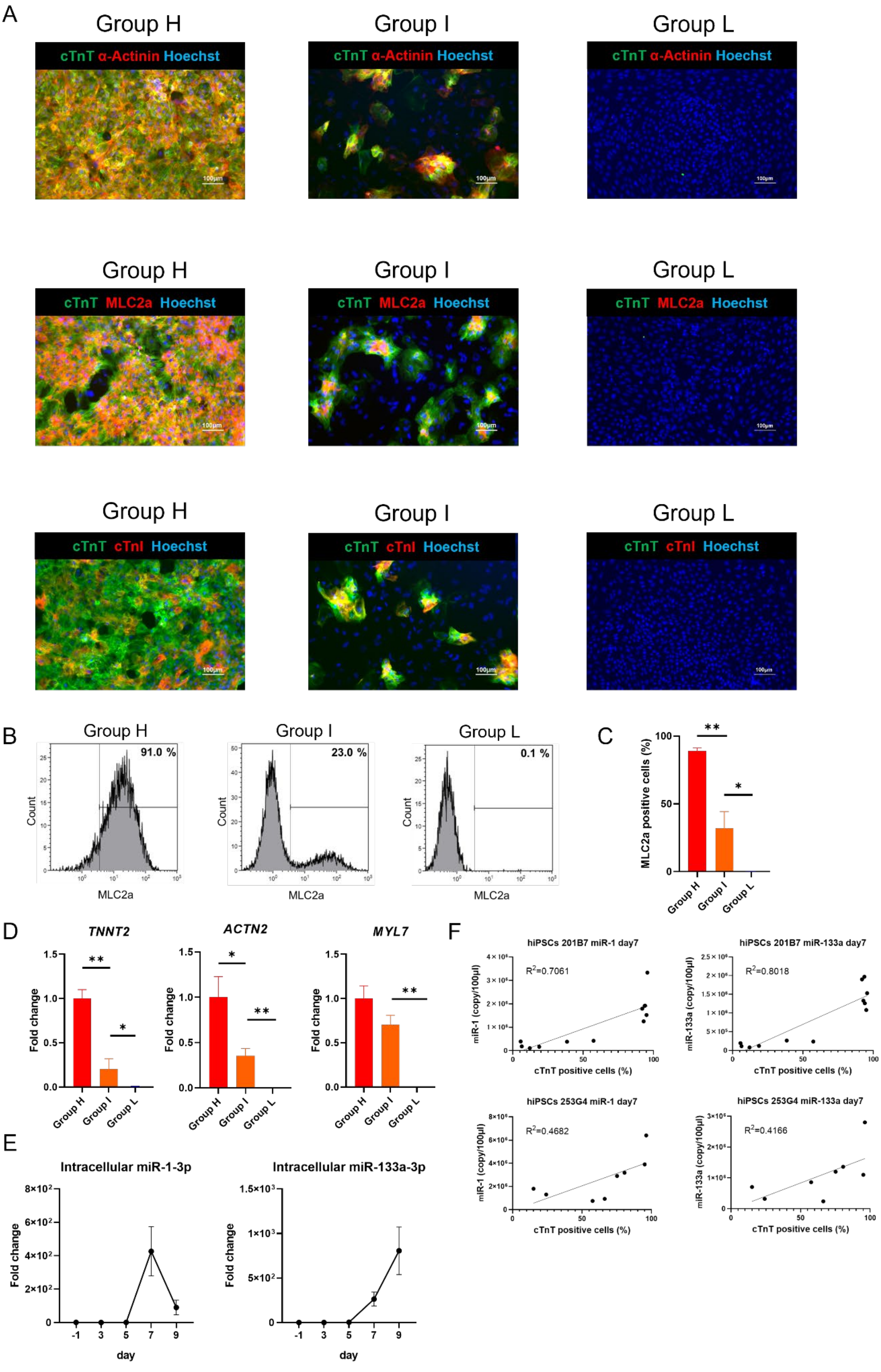

A
Group H
Group I
Group L
cTnT α-Actinin Hoechst
cTnT MLC2a Hoechst
cTnT cTnI Hoechst
100μm
B
91.0 %
23.0 %
0.1 %
Count
MLC2a
C
MLC2a positive cells (%)
D
TNNT2
ACTN2
MYL7
Fold change
E
Intracellular miR-1-3p
Intracellular miR-133a-3p
Fold change
day
F
hiPSCs 201B7 miR-1 day7
R^2=0.7061
hiPSCs 201B7 miR-133a day7
R^2=0.8018
hiPSCs 253G4 miR-1 day7
R^2=0.4682
hiPSCs 253G4 miR-133a day7
R^2=0.4166
miR-1 (copy/100μl)
miR-133a (copy/100μl)
cTnT positive cells (%)

Figure S3. Verification of the usefulness of miR-1-3p and miR-133a-3p for monitoring cardiac differentiation, related to Figure 3.

(A) Immunostaining for α-Actinin, MLC2a or cTnI (red), cTnT (green), and Hoechst (blue) in Groups H, I, and L on day 10 differentiated from hiPSCs (253G4). Scale bar, 100 µm.

(B and C) Flow cytometry analysis for MLC2a-positive cells in Groups H, I, and L on day 10 (n = 4 independent experiments for the 253G4 cell line).

(D) RT-qPCR analysis of the relative gene expression of *TNNT2*, *ACTN2,* and *MYL7* in Groups H, I, and L on day 10 (n = 4 independent experiments for 253G4 cell lines).

(E) miR-1-3p and miR-133a-3p expression in the cells during cardiac differentiation from hiPSCs (n = 4 independent experiments for 253G4 cell lines).

(F) Correlation between the percentage of cardiac troponin T-positive cells on day 10 and the amount of miR-1,133a-3p in the culture supernatant on day 7. R^2: Coefficient of determination.

Data are presented as mean ± SD; *p < 0.05; **p < 0.01. All p values are determined with a ratio-paired *t*-test.

Figure S4

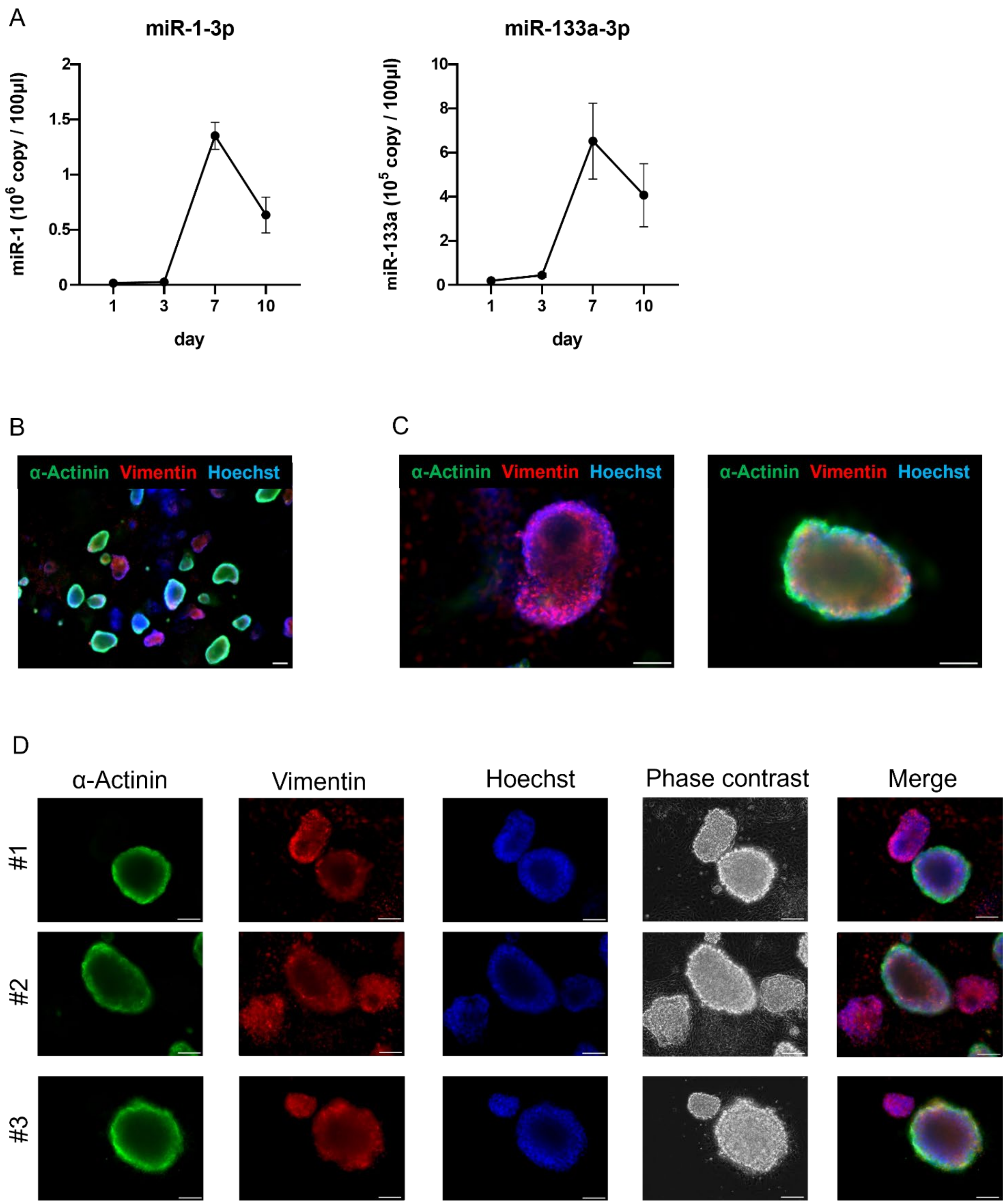

A
miR-1-3p
miR-1 (10^6 copy / 100µl)
2
1.5
1
0.5
0
1
3
7
10
day
miR-133a-3p
miR-133a (10^5 copy / 100µl)
10
8
6
4
2
0
1
3
7
10
day
B
α-Actinin Vimentin Hoechst
C
α-Actinin Vimentin Hoechst
α-Actinin Vimentin Hoechst
D
α-Actinin
Vimentin
Hoechst
Phase contrast
Merge
#1
#2
#3

Figure S4. Application of cardiac differentiation monitoring by measuring miR-1-3p and miR-133a-3p levels in culture supernatant to 3D culture, related to Figure 3.

(A) miR-1-3p and miR-133a-3p levels in the culture supernatant during cardiac differentiation from hiPSC-CMs in 3D culture method using bioreactors (n = 3 independent experiments for 253G4 cell lines).

(B) Immunostaining of multiple cell aliquots differentiated from hiPSCs (253G4) with α-Actinin (Green), Vimentin (red), and Hoechst (blue) collected when cultured in the same bioreactor for 3D culture. Scale bar, 200 μm.

(C and D) Enlarged images of the part of Figure S2. Scale bar, 100 μm.

Data are presented as mean ± SD.

Figure S5

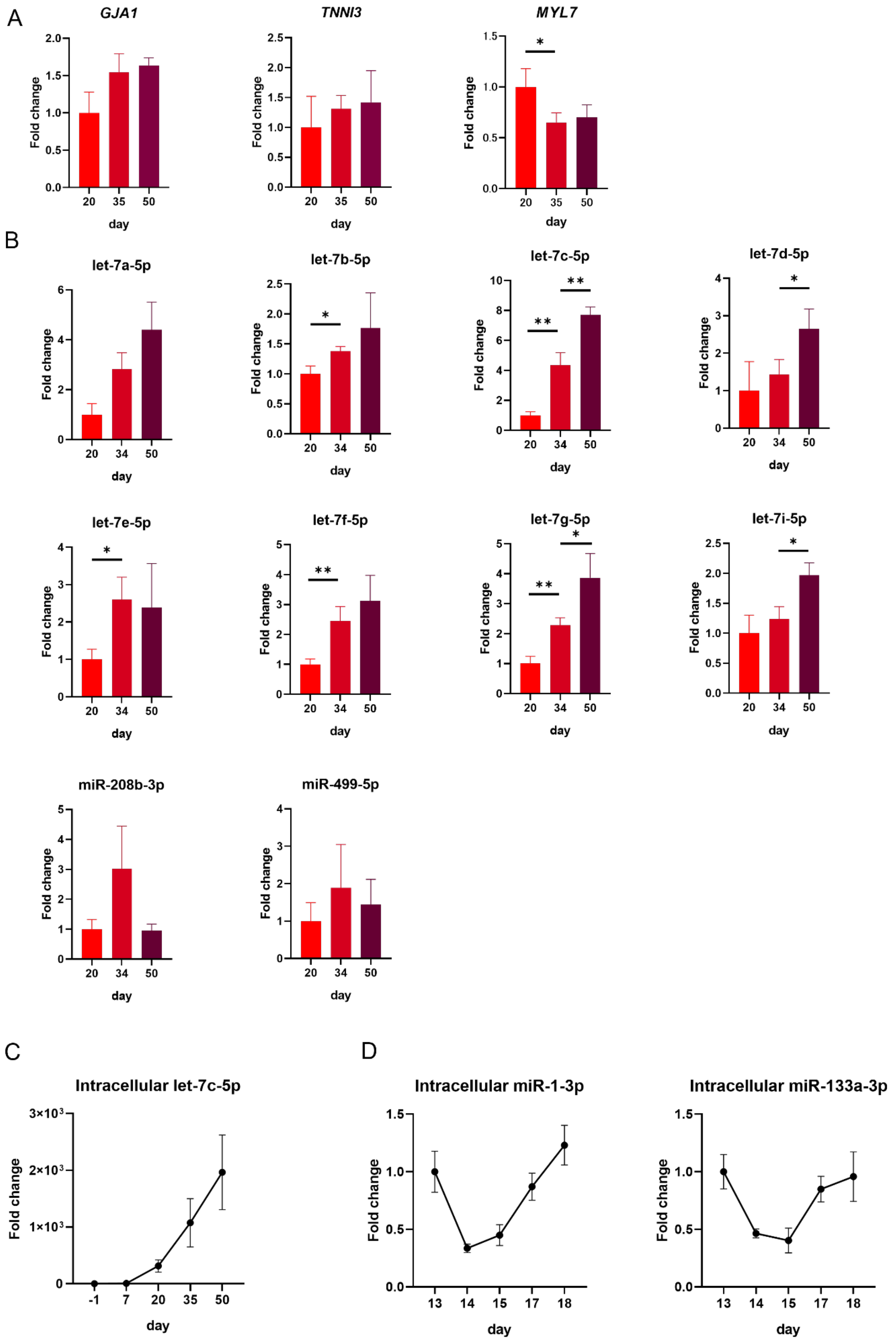
A
GJA1
TNNI3
MYL7
Fold change
day
B
let-7a-5p
let-7b-5p
let-7c-5p
let-7d-5p
let-7e-5p
let-7f-5p
let-7g-5p
let-7i-5p
miR-208b-3p
miR-499-5p
C
Intracellular let-7c-5p
D
Intracellular miR-1-3p
Intracellular miR-133a-3p

Figure S5. miRNAs that significantly increase in the supernatant during cardiac maturation, related to Figure 5.

(A) RT-qPCR analysis of the relative expression of *MYL7*, *GJA1*, and *TNNI3* in hiPSC-CMs on days 20, 35, and 50 measured by (n = 4 independent experiments for 253G4 cell lines).

(B) The fold change in the level of each miRNA in the culture supernatant during long-term culture of hiPSC-CMs on days 20, 34, and 50 (n = 3 independent experiments for the 253G4 cell lines).

(C) let-7c-5p expression in the cells during cardiac differentiation and maturation from hiPSCs (n = 4 independent experiments for 253G4 cell lines).

(D) miR-1-3p and miR-133a-3p expression in the cells during cardiac purification step (n = 3 independent experiments for 253G4 cell lines).

Data are presented as mean ± SD; $*p < 0.05$; $**p < 0.01$. All p-values are determined with a ratio-paired *t*-test.

Figure S6

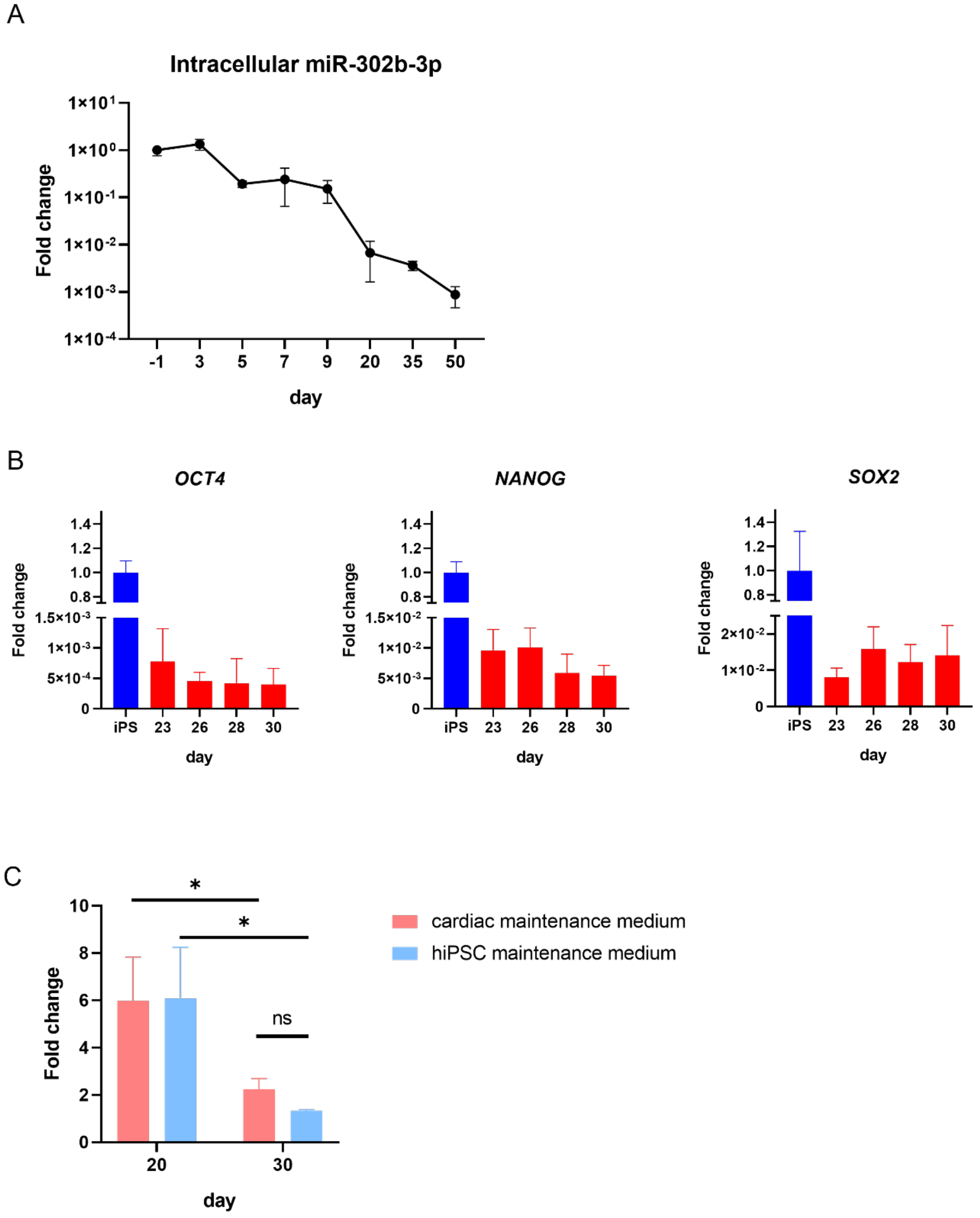
A
Intracellular miR-302b-3p
Fold change
1×10^1
1×10^0
1×10^-1
1×10^-2
1×10^-3
1×10^-4
-1 3 5 7 9 20 35 50
day
B
OCT4
NANOG
SOX2
Fold change
1.4
1.2
1.0
0.8
1.5×10^-3
1×10^-3
5×10^-4
0
1.5×10^-2
1×10^-2
5×10^-3
2×10^-2
iPS 23 26 28 30
day
C
10
8
6
4
2
0
Fold change
*
*
ns
20 30
day
cardiac maintenance medium
hiPSC maintenance medium

Figure S6. **Verification of the usefulness of miR-302b-3p for the detection of residual undifferentiated hiPSCs in hiPSC-CM cultures, related to Figure 6.**

(A) miR-302b-3p expression in the cells during cardiac differentiation and maturation from hiPSCs (n = 4 independent experiments for 253G4 cell lines).

(B) RT-qPCR analysis of the relative expression of *OCT4, NANOG,* and *SOX2* in pure hiPSCs and hiPSC-CMs on day 23, 26, 28, and 30 (n = 3 independent experiments for 253G4 cell lines).

(C) Intracellular expression of miR-302b-3p in hiPSC-CMs cultured under cardiac maintenance conditions and hiPSC maintenance conditions.

Data are presented as mean ± SD; *p < 0.05; **p < 0.01. All p-values are determined with a ratio-paired *t*-test.

Experimental procedures

Cell lines

Human induced pluripotent stem cell (hiPSC) lines (253G4 and 201B7) were obtained from the Center for iPS Cell Research and Application (CiRA), Kyoto University. Clinical-grade HLA homozygous hiPSC line (QHJI14s04) was also obtained from CiRA (Yoshida et al., 2023). The HEK293T cell line was obtained from RIKEN BioResource Research Center.

Maintenance of hiPSC lines

hiPSCs were maintained on Matrigel (Corning, 354230) or iMatrix511 (Nippi, NP892-011) coated plates in animal-free and chemically-defined hPSC maintenance medium (StemFit AS103C, Ajinomoto) (Kameda et al., 2022; Someya et al., 2021; Tohyama et al., 2017). Cells were routinely passaged every 5 or 6 days. After being washed with D-PBS (FUJIFILM Wako Pure Chemical, 045-29795), cells were dissociated using StemPro Accutase (Thermo Fisher Scientific, A1110501). Dissociated cells were collected in clinical-grade hiPSC maintenance medium, StemFit AS103C, with 10 µM of CultureSure Y-27632 (FUJIFILM Wako Pure Chemical, 034-24024). Following centrifugation (300 × g for 4 min), supernatant aspiration, and addition of hPSC maintenance medium with Y-27632, the number of cells was counted with a Vi-CELL XR (Beckman Coulter), and about 1×10^5 cells were then seeded onto a Matrigel- or iMatrix511-coated 10 cm plate. Media were changed every other day using the hPSC maintenance medium.

Maintenance of HEK293T cells

HEK293T cells were maintained in DMEM (Gibco, 11885) supplemented with 10% fetal bovine serum (FBS) (Biowest, S1560-500) on 0.1% gelatin-coated plates. HEK293T cells were routinely passaged every 3 or 4 days using a similar procedure to the passaging of hiPSCs mentioned above but using DMEM plus 10% FBS.

Production of hiPSC-CMs and supernatant sampling

hiPSC-derived cardiomyocytes (hiPSC-CMs) were generated as previously described (Tanosaki et al., 2022; Tohyama *et al.*, 2017; Tohyama et al., 2016). Briefly, 4 days before day 0 hiPSCs were passaged onto Matrigel- or iMatrix511-coated plates and incubated with clinical-grade hPSC maintenance medium for 4 days to reach 90–100% confluent. On day 0, cells were washed with D-PBS and incubated in animal-free and chemically-defined cardiac differentiation medium (StemFit AS301, Ajinomoto) or RPMI1640 (FUJIFILM

Wako Pure Chemical, 189-02025) supplemented with 2% B27 supplement without insulin (Thermo Fisher Scientific, A1895601), 6 μM CHIR99021 (FUJIFILM Wako Pure Chemical, 034-23103), and 1 ng/mL bone morphogenic protein 4 (BMP4) (R&D Systems, 314-BP) for 1 day. On day 1, cells were washed with D-PBS and incubated in clinical-grade StemFit AS301 or RPMI1640 supplemented with B27 supplement without insulin. The same procedure was followed on day 3 but 5 μM IWR-1 (Sigma-Aldrich, I0161-25MG) was added. On day 5 or 6, the cells were washed and the medium was replaced with clinical-grade StemFit AS301 or RPMI1640 supplemented with B27 supplement without insulin. On day 7, cells were incubated in MEMα (Thermo Fisher Scientific, 12571-048) supplemented with 5% FBS (SAFC Bioscience Inc.,12003C or Biowest, S1560-500) and 2 mM sodium pyruvate (Sigma, S8636-100ML) for maintenance of hiPSC-CMs. On days 8–10, the cells were washed with D-PBS and dissociated using 0.25% Trypsin/1 mM EDTA. Cells were collected and resuspended in MEMα plus 5% FBS and counted using Vi-CELL (Beckman Coulter). Cells were plated in plates coated with collagen type I (AGC TECHNO GLASS, 4020-010) or iMatrix-221 (Nippi, NP892-061). On day 13, the culture medium was replaced with glucose and glutamine-free medium supplemented with 4 mM lactate, animal-free and chemically-defined cardiac purification medium (StemFit AS501, Ajinomoto) for metabolic selection (Tohyama *et al.*, 2016; Tohyama et al., 2013). Four days after replacing the medium with clinical-grade cardiac purification medium (StemFit AS501, Ajinomoto), the culture medium was replaced by MEMα plus 5% FBS. On day 17 or later, cardiomyocytes were used for experiments. In the experiment to measure the amount of miR-489-3p, miR-1-3p, and miR-133a-3p secreted during the differentiation process, medium exchange and supernatant collection were performed from day −1 to day 9. In this differentiation process, we set three groups based on mesoderm and cardiac differentiation efficiency, high (Group H), intermediate (Group I), and low (Group L). In Group H, we produced hiPSC-derivatives with CHIR99021/BMP4 and IWR1. In Group I, we produced them with CHIR99021/BMP4 and without IWR1. In Group L, we produced them without CHIR99021/BMP4. In this experiment, the supernatant was collected at the time of the medium exchange. In collecting supernatant samples for miRNA microarray during the differentiation process, medium exchange and supernatant collection were performed as shown in **Figure 1A**. For the 3D culture method, 4×10^6 hiPSCs in 25 mL of hPSC maintenance medium with 10 μM Y-27632 were incubated in a single-use bioreactor (Biott, BWV-S03A) rotating at 60 rpm for 4 days before day 0, and hiPSC-CMs were subsequently induced following the same protocol as the 2D culture system.

Production of endothelial cells from hiPSCs

Briefly, 4 days before day 0 hiPSCs were passaged onto Matrigel- or iMatrix511-coated plates and incubated with hPSC maintenance medium for 4 days to reach 90–100% confluence. On day 0, cells were washed with D-PBS and incubated in RPMI1640 supplemented with 2% B27 supplement without insulin, 2 mM GlutaMAX (Gibco, 35050061), 6 μM CHIR99021, and 10 ng/mL BMP4 for 1 day. On day 1, cells were washed with D-PBS and incubated in RPMI1640 supplemented with B27 supplement without insulin, 2 mM GlutaMAX, and 10 μg/ml bFGF (Ajinomoto). The same procedure was followed on day 3. On day 4, the medium was replaced with RPMI1640 supplemented with B27 supplement without insulin, GlutaMAX, and 100 μg/mL VEGF (PeproTech, 100-20). On day 5, the cells were washed with D-PBS and dissociated using 0.25% Trypsin/1 mM EDTA and passaged onto Matrigel- or iMatrix511-coated plates and incubated with RPMI1640 supplemented with B27 supplement without insulin, GlutaMAX and 100 μg/mL VEGF. On days 6 and 8, the medium was replaced with the same as before.

Production of neural cells from hiPSCs

Ectodermal cells (Neural cells) were induced from hiPSCs using STEMdiff SMADi Neural Induction Kit (STEMCELL ST-08581). Briefly, hiPSCs were passaged onto Matrigel- or iMatrix511-coated plates at the density of 2.0-2.5 $\times 10^5$ cells/cm^2. The cells were then incubated with attached medium supplemented with 10 μM Y27632. The medium was changed to the same medium without Y27632 the next day and 2 days later. After 3 days, the cells were fixed in 4% paraformaldehyde (Muto Pure Chemicals, 33111) for 15–20 min and Immunostaining with Nestin was performed following the immunostaining protocol described below.

Immunofluorescence staining

Cells were washed once with D-PBS and fixed in 4% paraformaldehyde (Muto Pure Chemicals, 33111) for 15–20 min. Cells were then washed with D-PBS, and treated with 0.1% Triton X (Sigma-Aldrich, T9284) for 5–15 min. Cells were then washed with PBS supplemented with 0.05% Tween-20 (Thermo Fisher Scientific, 28360) (PBS-T) and treated with a blocking solution of ImmunoBlock (KAC, CTKN001) for 1 h at room temperature or overnight at 4°C. Following primary incubation with a primary antibody diluted in ImmunoBlock overnight at 4°C, cells were washed twice with D-PBS and co-incubated with a secondary antibody diluted in ImmunoBlock at room temperature for 2 h. Cells were washed twice more with D-PBS and treated with 5 μg/mL Hoechst 33342 (Thermo Fisher Scientific, H3570) at room temperature for 1 h. Finally, cells were washed twice with D-PBS and incubated in ImmunoBlock until staining was examined using BZ-X710

(Keyence). Immunofluorescence staining of cultured cells was performed using the following primary antibodies; anti-Brachyury T (Abcam, ab209665;1:200), anti-MIXL1 (Proteintech Group Inc, 22772-1-AP; 1:100), anti-EOMES (Abcam, ab216870; 1:100), anti-CD31(Abcam, ab28364; 1:50), and anti-Nestin (Abcam, ab 105389; 1:200), anti-OCT-3/4 (Abcam, ab200834; 1:200), anti-SSEA4 (Chemicron, MAB4304; 1:100), anti-cardiac Troponin T (Abcam, ab45932; 1:800, or Neomarkers, MS-295-P; 1:200), anti-cardiac Troponin I (Abcam, ab52862; 1:100), anti-α-Actinin (Abcam, ab137346; 1:100), anti-N-cadherin (Invitrogen, 333900; 1:100), anti-Connexin 43 (Sigma-Aldrich, C6219; 1:100), anti-MLC2a (Synaptic Systems C156F5 311011; 1:100), anti-MLC2v (Abcam, Cambridge, USA, ab 79935; 1:100), and anti-Vimentin (Abcam, ab8069; 1:1000) and using the following secondary antibodies: anti-rabbit IgG or anti-mouse IgG conjugated with Alexa Fluor 488, Alexa Fluor 546, or Alexa Fluor 594 (all from Thermo Fisher Scientific; 1:200).

Colony formation assay for detection of residual undifferentiated hPSCs

The colony formation assay (CFA) was performed to confirm the absence of undifferentiated hiPSCs in the hiPSC-CMs used for experiments, following previous reports (Tano et al., 2014; Tohyama *et al.*, 2016). A total of 3×10^6 HEK293T cells or hiPSC-CMs were seeded onto 6-well plates coated with iMatrix-511; the group without hiPSC contamination served as a negative control, the group contaminated with 0.001% hiPSCs served as a positive control, and the sample to be verified was also seeded 3×10^6 cells/well. All groups were cultured in hPSC maintenance medium (StemFit AS103C, Ajinomoto) for 2 days. Subsequently, they were fixed with 4% paraformaldehyde, and OCT4 and SSEA4-positive colonies were detected via immunostaining. If a colony was detected in the positive control and not detected in the sample as well as the negative control, the contamination rate of residual undifferentiated hiPSCs in the sample is less than 0.001%.

Flow cytometry analysis

Cells were washed with D-PBS and incubated with 0.25% Trypsin/1 mM EDTA or StemPro Accutase (Thermo Fisher Scientific) for 5 min. Following collection of the dissociated cells, they were centrifuged (300 g × 4 min), the supernatant was aspirated and the cells were fixed by adding 4% paraformaldehyde for 20 min. D-PBS was then added to the cells, and following centrifugation (300 × g for 4 min) and supernatant aspiration, 0.1% Triton X was added for 5–15 min. Then, the cells were washed with PBS-T as mentioned above, dispensed into aliquot tubes with 100 μL of a solution containing ImmunoBlock with 2 μL of the specific antibody added

to each tube, and left in the dark for 15–20 min. Cells were washed with PBS, diluted in ImmunoBlock, and analyzed using a Gallios Flow Cytometer (Beckman Coulter). Antibodies used were: anti-REA Control FITC, human (Miltenyi Biotec, 130-118-354), anti-REA Control APC, human (Miltenyi Biotec, 130-118-546), anti-cardiac Troponin T (cTnT), human (Miltenyi Biotec, 130-119-575), anti-cardiac MLC2a, human (Miltenyi Biotec, 130-118-546), IgG1-PE Isotypic Control (IMMUNOTECH SAS, A07796), and anti-CD13-PE (IMMUNOTECH SAS, A07762).

RNA extraction and quantitative RT-PCR analysis

Total RNA samples were extracted using ReliaPrep™ RNA Cell Miniprep System (Promega) according to the manufacturer's instructions. The concentration and purity of the RNA were measured using an ND-1000 spectrophotometer (Nanodrop) and the cDNA was synthesized using the Superscript First Strand Synthesis System (Invitrogen). The reactions were incubated in Simpliamp (Applied Biosystems) in a 96-well plate according to the manufacturer's instructions. Quantitative PCR (qPCR) was performed using FastStart Essential DNA Probes Master (Roche). The reactions were incubated in a LightCycler® 96 System (Roche) in a 96-well plate at 95°C for 10 min, followed by 40 cycles of 95°C for 15 sec and 60°C for 1 min. The threshold cycle (Ct) was defined as the fractional cycle number at which the fluorescence passed the fixed threshold. The amount of mRNA was normalized against that of GAPDH mRNA. RT primers, PCR primers, and probes were purchased as TaqMan™ Gene expression Assays from ThermoFisher Scientific (*GAPDH*, Hs02758991; *TNNT2*, Hs00943911; *ACTN2*, Hs00153809; *MYL2*, Hs00166405; *MYL7*, Hs01085598; *TNNI3*, Hs0016957; *GJA1*, Hs00748445; *EOMES*, Hs00172872; *MIXL1*, Hs00430824; *MESP1*, Hs00251489; *NANOG*, Hs02387400; *OCT4*, Hs04260367; *SOX2*, and Hs04234836). In the TaqMan hPSC Scorecard Panel assay, according to the manufacturer's protocol, the cDNA was synthesized using the High-Capacity cDNA Reverse Transcription Kit with RNase Inhibitor (Applied Biosystems); PCR was performed using 2 × TaqMan Fast Advanced Master Mix (Applied Biosystems) and the reactions were incubated in StepOnePlus Real-Time PCR System (Applied Biosystems) in a 96-well plate. Analysis was performed by inputting data into the hPSC Scorecard analysis software (Thermo Fisher Scientific).

miRNA microarray analysis

Total RNA including miRNA was isolated from cell culture supernatant using a miRNeasy Serum/Plasma Kit (Qiagen, Valencia, CA). RNA quantity and quality were determined using an Agilent 2100 Bioanalyzer

(Agilent Technologies, Inc.). RNA was labeled with cyanine 3 (Cy3) using miRNA Complete Labeling and Hyb Kit (Agilent Technologies, Inc.) following the manufacturer's instructions. Briefly, RNA was dephosphorylated using Calf Intestinal Alkaline Phosphatase (CIP) Master Mix incubated at 37°C for 30 min. Dephosphorylated RNA was denatured with DMSO incubated at 100°C for 5 min and then immediately transferred to ice for 2 min. These products were mixed with a Ligation Master Mix for T4 RNA Ligase and Cy3-pCp (Cyanine 3-Cytidine biphosphate) and incubated at 16°C for 2 h. Labeled RNA was dried using a vacuum concentrator at 55°C for 1.5 h. Cy3-pCp-labeled RNA was hybridized on Agilent Human miRNA Microarray (Design ID: 070156) array at 55°C for 20 h. After washing, microarrays were scanned using an Agilent SureScan Microarray Scanner System (G4900DA). Intensity values for each scanned feature were quantified using Agilent Feature Extraction software version 12.1.1.1, which performs background subtractions. To identify miRNAs that are useful for monitoring mesoderm/cardiac differentiation and maturation processes, we evaluated the expression ratio of each miRNA at two specific time points. For the residual undifferentiated markers and the mesoderm/cardiac differentiation markers, we selected those with high expression ratios in the respective appropriate pair of samples and considered any reported significance as markers in the literature, if available. As criteria for selecting cardiac maturation markers, we considered not only a higher expression ratio in day 51 samples compared to day 9, day 21, and day 35 samples but also a consistent trend of either remaining constant or increasing monotonically over time.

Microarray analysis

Total RNA was isolated from cell culture supernatant using a ReliaPrep™ RNA Cell Miniprep System (Promega) according to the manufacturer's instructions. Total RNA quantity and quality were determined using NanoDrop One spectrophotometer (Thermo Fisher Scientific Inc.) and Agilent 2100 Bioanalyzer (Agilent Technologies, Santa Clara, CA). For cRNA amplification and labeling, Agilent Low Input Quick Amp Labeling Kit (Agilent Technologies) was used following the manufacturer's instructions. Briefly, total RNA was reverse-transcribed to double-strand cDNA using poly dT-T7 promoter primer at 65℃ for 10 min, then incubated at 40℃ for 2 h with 5X First Strand Buffer, 0.1 M DTT, 10 mM dNTP mix, and Affinity Script Rnase Block Mix. The Affinity Script enzyme was inactivated at 70℃ for 15 min. The cDNA products were used as templates for in vitro transcription to generate fluorescent cRNA. cDNA template were combined with 5x Transcription Buffer, 0.1 M DTT, NTP mix, T7 RNA polymerase, and Cyanine3-CTP, followed by incubation at 40℃ for 2 h. Labeled cRNAs were purified using Rneasy mini spin columns (Qiagen) and eluted in 30 μl

of nuclease-free water. The cRNA quantity and Cyanine-3 (Cy3) dye incorporation were determined using NanoDrop One spectrophotometer and Agilent 2100 Bioanalyzer. For each hybridization, 0.60 μg of Cy3 dye-labeled cRNA were fragmented and hybridized at 65℃ for 17 h using the Agilent SurePrint G3 Human GE v3 8x60K Microarray (Design ID: 072363). After washing, the microarrays were scanned using Agilent SureScan Microarray Scanner (G4900DA). Intensity values of each scanned feature were quantified using Agilent Feature Extraction software version 12.1.1.1, which performs background subtractions. We only used features that were flagged as no errors (Detected flags) and excluded features that were not positive, significant, uniform, above background, and saturated, and population outliers (Not Detected and Compromised flags). Quantile Normalization was performed using Agilent GeneSpring software version 14.9.1.

Calcium transients

To examine intracellular calcium transients, hiPSC-CMs were incubated in 1mM Cal 520, AM (AAT Bioquest, 21130) for 90 min at 37°C and analyzed with a BZ-X710 microscope and BZ-X800 Analyzer (Keyence) 30 min after room temperature incubation. The time to 50% decay was calculated from the decreasing limbs of the transients.

Statistical analysis

All statistical analysis was performed using Prism (GraphPad). All data are expressed as mean ± SD. All differences between the two group analyses were determined with the paired t-test. $P < 0.05$ was considered statistically significant.

REFERENCES

Kameda, K., Someya, S., Fujita, J., Fukuda, K., and Tohyama, S. (2022). Protocol for enhanced proliferation of human pluripotent stem cells in tryptophan-fortified media. STAR Protoc *3*, 101341. 10.1016/j.xpro.2022.101341.

Someya, S., Tohyama, S., Kameda, K., Tanosaki, S., Morita, Y., Sasaki, K., Kang, M.I., Kishino, Y., Okada, M., Tani, H., et al. (2021). Tryptophan Metabolism Regulates Proliferative Capacity of Human Pluripotent Stem Cells. iScience *24*, 102090. 10.1016/j.isci.2021.102090.

Tano, K., Yasuda, S., Kuroda, T., Saito, H., Umezawa, A., and Sato, Y. (2014). A novel in vitro method for detecting undifferentiated human pluripotent stem cells as impurities in cell therapy products using a highly efficient culture system. PLoS One *9*, e110496. 10.1371/journal.pone.0110496.

Tanosaki, S., Akiyama, T., Kanaami, S., Fujita, J., Ko, M.S.H., Fukuda, K., and Tohyama, S. (2022). Purification of cardiomyocytes and neurons derived from human pluripotent stem cells by inhibition of de novo fatty acid synthesis. STAR Protoc *3*, 101360. 10.1016/j.xpro.2022.101360.

Tohyama, S., Fujita, J., Fujita, C., Yamaguchi, M., Kanaami, S., Ohno, R., Sakamoto, K., Kodama, M., Kurokawa, J., Kanazawa, H., et al. (2017). Efficient Large-Scale 2D Culture System for Human Induced Pluripotent Stem Cells and Differentiated Cardiomyocytes. Stem Cell Reports *9*, 1406-1414. 10.1016/j.stemcr.2017.08.025.

Tohyama, S., Fujita, J., Hishiki, T., Matsuura, T., Hattori, F., Ohno, R., Kanazawa, H., Seki, T., Nakajima, K., Kishino, Y., et al. (2016). Glutamine Oxidation Is Indispensable for Survival of Human Pluripotent Stem Cells. Cell Metab *23*, 663-674. 10.1016/j.cmet.2016.03.001.

Tohyama, S., Hattori, F., Sano, M., Hishiki, T., Nagahata, Y., Matsuura, T., Hashimoto, H., Suzuki, T., Yamashita, H., Satoh, Y., et al. (2013). Distinct metabolic flow enables large-scale purification of mouse and human pluripotent stem cell-derived cardiomyocytes. Cell Stem Cell *12*, 127-137. 10.1016/j.stem.2012.09.013.

Yoshida, S., Kato, T.M., Sato, Y., Umekage, M., Ichisaka, T., Tsukahara, M., Takasu, N., and Yamanaka, S. (2023). A clinical-grade HLA haplobank of human induced pluripotent stem cells matching approximately 40% of the Japanese population. Med (N Y) *4*, 51-66.e10. 10.1016/j.medj.2022.10.003.
